# Semi-supervised emotion-driven music generation model based on category-dispersed Gaussian Mixture Variational Autoencoders

**DOI:** 10.1371/journal.pone.0311541

**Published:** 2024-12-30

**Authors:** Zihao Ning, Xiao Han, Jie Pan

**Affiliations:** 1 Communication University of China, Nanjing, China; 2 Nanjing University of Aeronautics and Astronautics, Nanjing, China; Bayer Crop Science United States: Bayer CropScience LP, UNITED STATES OF AMERICA

## Abstract

Existing emotion-driven music generation models heavily rely on labeled data and lack interpretability and controllability of emotions. To address these limitations, a semi-supervised emotion-driven music generation model based on category-dispersed Gaussian mixture variational autoencoders is proposed. Initially, a controllable music generation model is introduced, which disentangles and manipulates rhythm and tonal features, enabling controlled music generation. Building on this, a semi-supervised model is developed, leveraging a category-dispersed Gaussian mixture variational autoencoder to infer emotions from the latent representations of rhythm and tonal features. Finally, the objective loss function is optimized to enhance the separation of distinct emotional clusters. Experimental results on real-world datasets demonstrate that the proposed method effectively separates music with different emotions in the latent space, thereby strengthening the association between music and emotions. Additionally, the model successfully disentangles and separates various musical features, facilitating more accurate emotion-driven music generation and emotion transitions through feature manipulation.

## 1. Introduction

Music, as a significant form of artistic expression, reflects a series of human-specific cognitive patterns. Similar to natural language, music conveys content and emotions through sequential information. Unlike natural language, however, emotion is a crucial component of the information conveyed by music [1]. The emotions evoked by music are not only closely related to its inherent structure but are also induced by the sequential propagation of musical elements over time. Therefore, the automatic generation of music must not only adhere to the rules of music theory but also incorporate emotional elements to inspire creativity [2]. Emotion-driven music generation has wide applications, including the creation of background music for specific scenes in films or games, and in music-based psychotherapy [3].

In recent years, significant advancements have been made in automatic music generation technology, with numerous generative models capable of effectively learning the structural features of music to produce rich musical samples [4]. However, fewer studies have focused on incorporating emotional elements as a condition for music generation. A typical approach to emotion-driven music generation involves using sequence-to-sequence (Seq2Seq) models, such as Recurrent Neural Networks (RNNs) and Transformers, to simultaneously encode both music sequences and emotion labels [5]. This allows the models to learn the feature representations of specific emotions, which are then decoded to generate music corresponding to the targeted emotions. Alternatively, emotion classifiers may be used post-generation to guide the emotional content of the music. Zhao et al. [6] constructed an emotion-driven music generation model using a biaxial LSTM architecture [7] combined with a Lookback module. Ferreira et al. [8] encoded music as a series of textual events and utilized multiplicative LSTM networks to create music with specific emotional attributes. To further enhance the model’s ability to capture long-term dependencies in the structural context of music, pre-trained Transformer models were employed in the works of Hung et al. [9] and Sulun et al. [10] to achieve emotion-driven music generation. In these models, emotional information, like the input musical events, is embedded as tokens within the model. To obtain better semantic representations of emotions, some studies utilized Variational Autoencoders (VAEs) [11] to learn emotion label embeddings and model the latent space of the data [12]. This approach maps the data distribution into a low-dimensional continuous space, from which diverse and continuous samples can be generated. Grekow et al. [13] proposed using Conditional Variational Autoencoders (CVAE) to generate music with specific emotions, leveraging Gated Recurrent Units (GRU) [14] to construct the encoder and decoder. Huang et al. [15] further combined CVAE with GANs to generate more accurate emotional music. In this model, a discriminator distinguishes the authenticity of the music generated by the CVAE decoder, while an additional emotion classifier determines the emotional category of the output music, thus aligning the generated music more closely with the target emotion. However, the aforementioned methods rely on fully supervised training and generate emotion-specific music directly based on labels, which presents two significant challenges. First, fully supervised training excessively depends on labeled data, yet the music domain lacks abundant standardized emotional datasets. Moreover, emotion is highly subjective, leading to considerable label noise during data annotation. Second, emotion labels alone are insufficient to fully capture the emotional characteristics of music. Generating music directly from labels overlooks the intricate relationships between emotions and musical elements, rendering the generated music less interpretable in terms of its underlying emotional representation.

Recent research, such as Music FaderNets [16], utilized Gaussian Mixture Variational Autoencoders (GMVAE) [17] in a semi-supervised approach to learn latent space representations of note and rhythm features along the Arousal dimension of Russell’s two-dimensional emotion space [18]. These features were then employed to control the Arousal dimension, enabling the generation of emotion-specific music. GMVAE extends the unimodal Gaussian distribution in VAE to a mixture of Gaussians with multiple components, allowing the latent space to be partitioned into distinct clusters. This enables direct inference of data categories from latent variables without the need for additional neural networks to learn category information, demonstrating high performance in semi-supervised generative tasks. Luo et al. [19] proposed a GMVAE-based audio music generation model that disentangled and learned latent spaces for different categories of pitch and timbre. When generating music with specific pitch or timbre, the corresponding latent variables were resampled from the target category’s latent space and decoded into the output. Tan et al. [20] also employed GMVAE in the Music FaderNets model to achieve controllable generation of MIDI music notes and rhythms. However, GMVAE has certain limitations, including the potential for mode collapse [21]. When data is overly similar, the Gaussian components may not separate well during training, making it difficult to distinguish between different categories, thus impacting the quality of the learned representations and generated outputs. Furthermore, most existing VAE-based music generation models rely on Recurrent Neural Networks (RNNs) as encoders and decoders [22]. Given that music is a long-sequence data type, RNNs are limited in their ability to model such sequences, often leading to the loss of contextual dependencies and issues like gradient vanishing or explosion.

To address these challenges, this paper proposes a semi-supervised emotion-driven music generation model based on Category-Dispersed Gaussian Mixture Variational Autoencoders (CDGMVAE). This model is designed to learn the influence of different musical features on emotions, generate music with long-term dependency structures and specific emotions, and achieve emotion transitions by manipulating musical features. The specific contributions of this paper are as follows:

➢ A semi-supervised emotion-driven music generation model based on GVAE with variance penalties and mutual information enhancement is proposed. This model ensures better separation of different emotional music in the latent space, strengthens the correlation between music and emotional information, aligns the generated music more closely with the target emotion, and improves the robustness and generalization ability of the semi-supervised model.
➢ A feature disentanglement mechanism constrained by independent encoder constraints and generative adversarial loss functions is proposed to separate and learn the latent variable representations of rhythm and tonal features from music sequences. By manipulating these two feature representations, the generation and transformation of specific emotional music can be achieved.
➢ The Transformer-XL network [23] is introduced as the encoder and decoder for GMVAE. Its segment-level recurrence mechanism and relative positional encoding effectively capture the longer contextual dependencies in music sequences and enhance the model’s ability to focus on different features, thereby improving the model’s expressiveness.

## 2. Disentangled controllable generation with Variational Autoencoders

Variational Autoencoders (VAEs) combine latent variable models with deep generative models, offering powerful representation and generation capabilities. By incorporating a disentanglement representation mechanism, VAEs can learn representations of multiple features, imbuing latent variables with more meaningful semantic interpretations. Due to the manipulability of the latent space, VAEs are effective models for achieving controllable music generation. Building on the principles of the VAE model, this paper proposes a controllable music generation model that manipulates latent variable representations to alter the structure of music, referred to as Control-VAE. The model structure is illustrated in **Fig 2**, comprising an encoder, a latent space, and a decoder. Given an input sequence **x** = [*Bar*_1_,*Bar*_2_,⋯,*Bar*_*T*_] containing *T* consecutive bars of music, the encoder *q*_*ϕ*_(**z**|**x**) learns the latent variable representations **z** of each bar through posterior inference. These representations are then used by the decoder *p*_*θ*_(**x**|**z**) to reconstruct the input data, producing a new sample $\tilde{x}$. The model can be optimized by maximizing the evidence lower bound (ELBO) of the log-likelihood function *p*(**x**), as shown in Eq (1):

$$\log\;p(x)\geq E_{q_{\phi }(z|x)}[\log\;p_{\theta }(x|z)]-D_{KL}[q_{\phi }(z|x)\|p(z)]={ℒ}_{ELBO}$$ (1)

where E[⋅] denotes the expectation, representing the need to maximize the probability of generating real data, which can be achieved by minimizing the reconstruction loss between the input |**x** and the output $\tilde{x}$. The term *D*_*KL*_[⋅‖⋅] represents the KL divergence that needs to be minimized between the latent distribution *q*_*ϕ*_(**z**|**x**) and the prior Gaussian distribution *p*(**z**).

**Fig 1:**
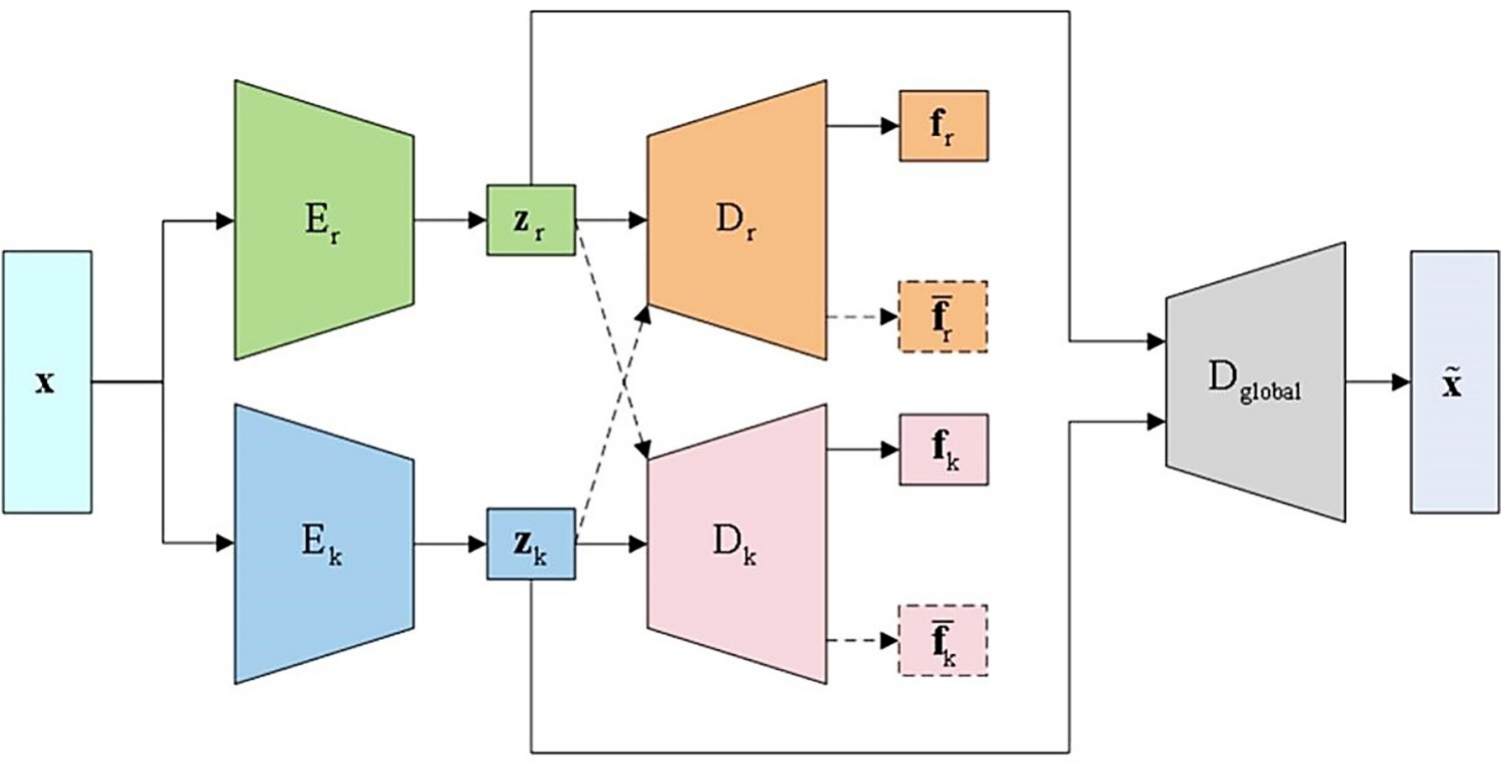
Structure of music feature disentanglement.

To achieve disentangled and controllable generation of rhythm and tonal features in music, this paper proposes a feature disentanglement mechanism constrained by independent encoders and adversarial loss functions, as depicted in **Fig 1**. Considering the rhythm features **f**_*r*_ and tonal features **f**_*k*_ of music, two identical encoder networks *E*_*r*_ and *E*_*k*_ were trained separately to learn the latent variable representations **z**_*r*_ and **z**_*k*_ of the rhythm features **f**_*r*_ and tonal features **f**_*k*_ from the original music sequence. Two separate local decoders *D*_*r*_ and *D*_*k*_ were then used to predict the rhythm features **f**_*r*_ and tonal features **f**_*k*_, with the training objective being to minimize the error between the real features and the predicted features. Additionally, adversarial training is incorporated into the model based on the concept of GANs. By feeding inputs **z**_*r*_ and **z**_*k*_ as the input to *D*_*r*_ and *D*_*k*_, incorrect information is fed back to each decoder to produce false predictions ${\bar{f}}_{r}$ and ${\bar{f}}_{k}$, thereby enabling *D*_*r*_ and *D*_*k*_ F to adversarially counteract each other during training. This ensured that only the correct feature predictions were made based on the corresponding latent variable inputs, effectively removing irrelevant feature information from each latent variable. Finally, the two latent variables were merged (i.e., **z** = *Concat*[**z**_*r*_,**z**_*k*_]) and input into the global decoder *D*_*global*_ to generate the complete music sequence, which is optimized using the ELBO. According to the proposed method, the objective loss function of the Control-VAE model can be defined as:

$${ℒ}_{\mathrm{ELBO}}=E_{q_{\phi_{r}}(z_{r}|x)q_{\phi_{k}}(z_{k}|x)}[\log\;p_{\theta }(x{|z}_{r},z_{k})]-{ℒ}_{KL}^{i}+{ℒ}_{\mathrm{Dis}}$$ (2)

where ${ℒ}_{KL}^{i}=D_{KL}[q_{\phi_{i}}(z_{i}|x)\|p(z_{i})]$, *i* represent the rhythm or tonal features, and ℒ_Dis_ represents the disentanglement loss, defined as follows:

$$\begin{array}{l} {ℒ}_{\mathrm{Dis}}=E_{q_{\phi_{r}}(z_{r}|x)q_{\phi_{k}}(z_{k}|x)}[\log\;p_{\varphi_{r}}(f_{r}|z_{r})p_{\varphi_{k}}(f_{k}|z_{k})]\\ \;+E_{q_{\phi_{r}}(z_{r}|x)q_{\phi_{k}}(z_{k}|x)}[\log(1-p_{\varphi_{r}}(f_{r}|z_{r}))(1-p_{\varphi_{k}}(f_{k}|z_{k}))] \end{array}$$ (3)

where the first term represents the loss when correctly predicting the features, and the second term represents the adversarial loss.

## 3. Category-dispersed Gaussian mixture variational autoencoder

**Fig 2:**
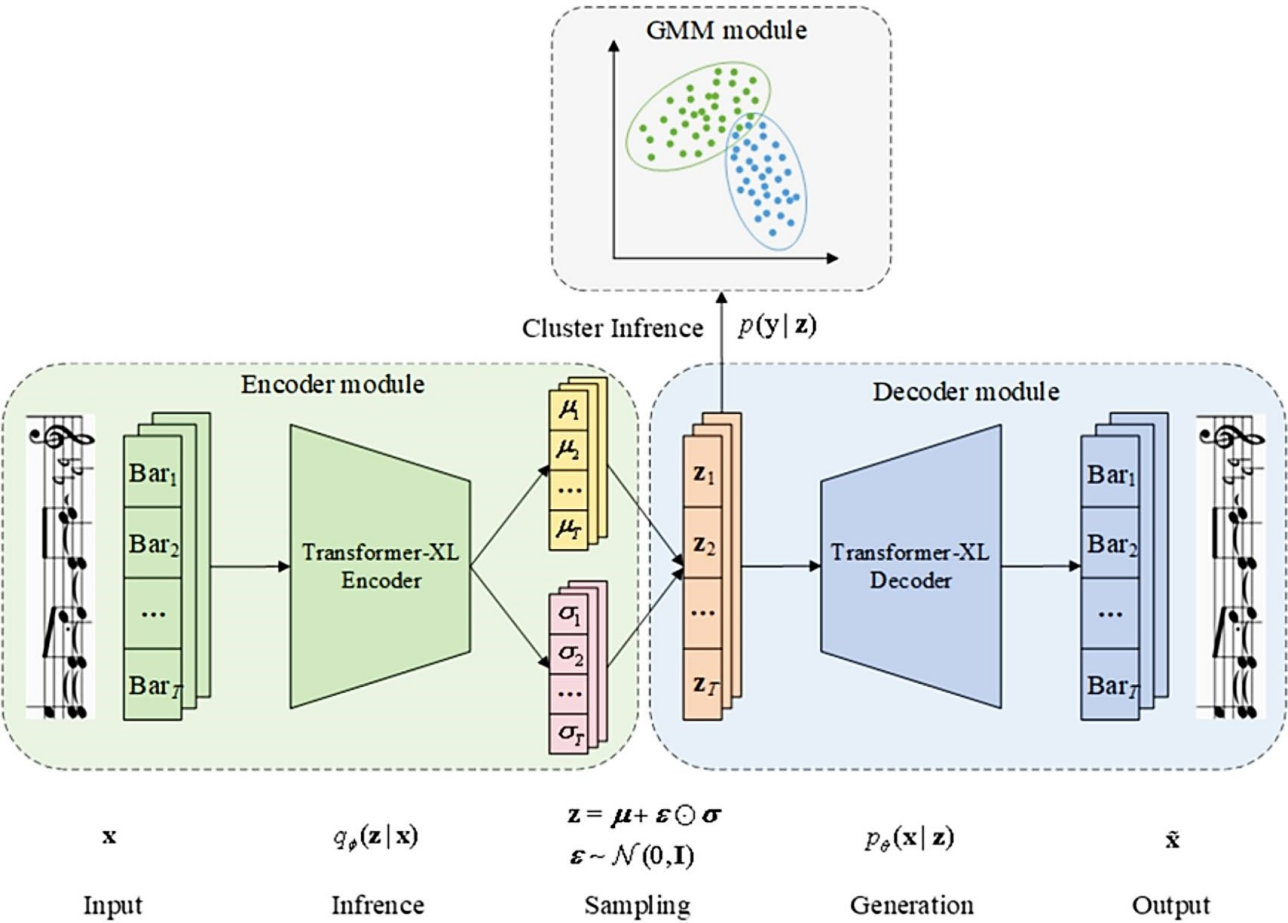
Schematic of the CDGMVAE model structure.

The structure of our method CDGMVAE is illustrated in **Fig 2** and comprises three main modules: (1) The encoder module, which receives the music input sequence segmented by bars and uses a Transformer-XL encoder to learn the mean and variance of the latent distribution; (2) The decoder module, which reparametrizes the latent variable representations sampled from the latent distribution and uses a Transformer-XL decoder to reconstruct the music sequence; and (3) The Gaussian mixture module, which employs a Gaussian mixture model (GMM) [78] to learn the categorical information of latent variables via semi-supervised learning, thereby clustering the music data by emotion.

### 3.1 Semi-supervised Gaussian mixture variational autoencoder

GMVAE integrates a VAE with a GMM, enabling the extension of the continuous latent space of a VAE into a discrete space with multiple Gaussian components. Each Gaussian component represents a cluster that contains latent variable representations of the same category. The CDGMVAE model leverages this property to infer the emotional category of the latent variable representations of music features using a semi-supervised approach, projecting them onto the corresponding Gaussian components. During the generation phase, latent variables are sampled from the Gaussian component of the target category, and the corresponding emotional music is generated by decoding these latent variables. The semi-supervised GMVAE can guide the model to learn the feature representations and boundaries of each category using a small amount of labeled data as supervision signals, while the unlabeled data provides a broader data distribution. By leveraging the labeled data, similar samples can be grouped into the same category without the need to introduce additional neural network classifiers.

**Fig 3:**
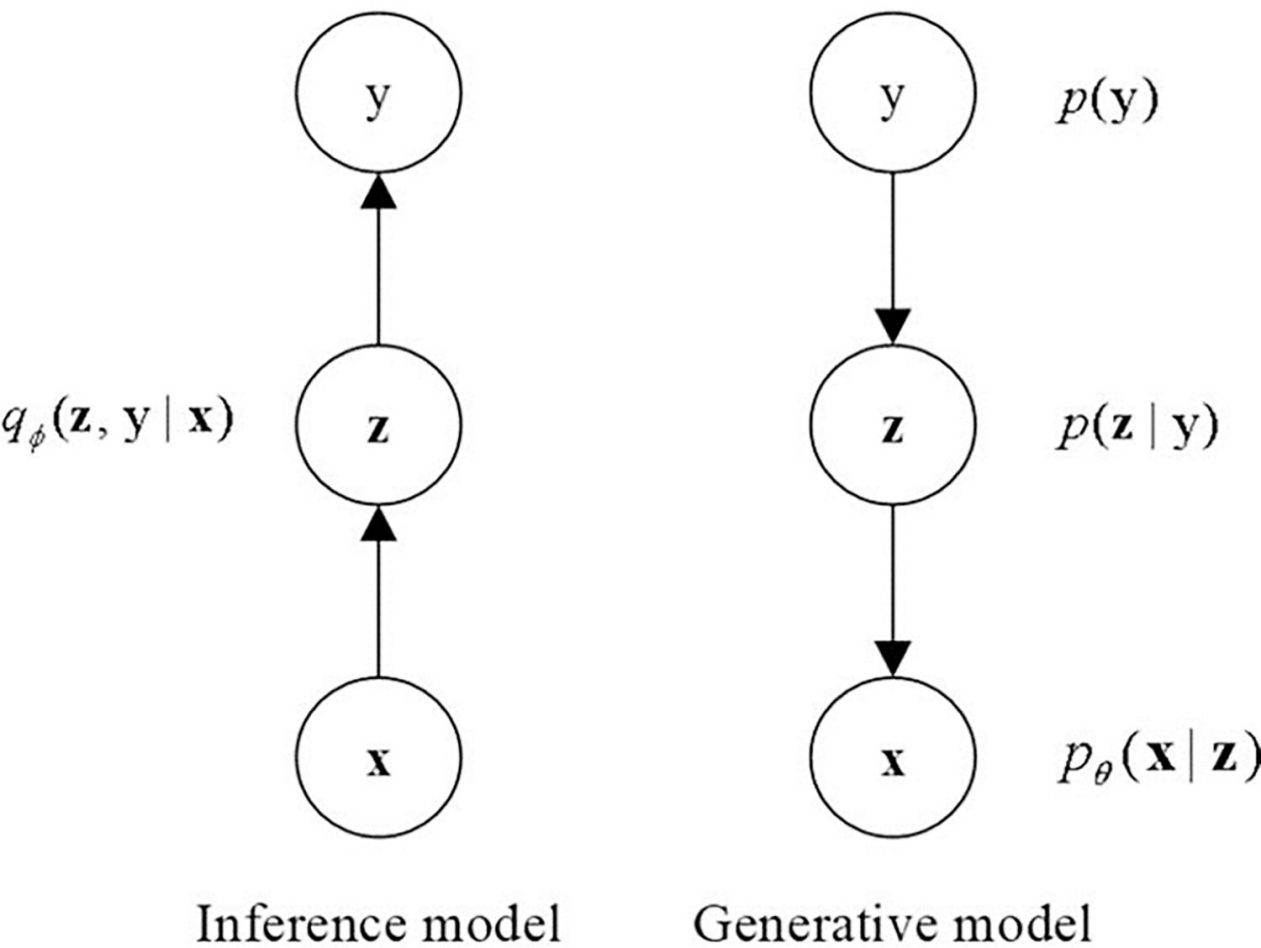
Probabilistic graphical model of GMVAE.

In implementation, the GMVAE can be divided into an inference network and a generative network, with its probabilistic graphical model illustrated in Fig 3. For the inference network, given the music input sequence *x*, the encoder learns the latent variable representations *z* of any feature and introduces a classification variable *y* to infer the emotional category *z*. For the generative network, latent variables *z* are sampled from the Gaussian component of the target category based on the classification variable *y*, and the decoder reconstructs the input data *x* based on these latent variables. The generative process can be defined by the joint probability distribution as shown in Eq (4):

$$p_{\theta }(x,z,y)=p_{\theta }(x|z)p(z|y)p(y)$$ (4)

where p(y)=Cat(y|1/K) represents the categorical distribution with *K* categories. $p(z|y)=N(z|\mu_{y},\sigma_{y}^{2})$ denotes the latent distribution of a specific Gaussian component within a category, characterized by a learnable mean *μ*_*y*_ and variance $\sigma_{y}^{2}$. *q*_*θ*_(*x*|*z*) is a neural network parameterized by *θ*, which is responsible for decoding and generating sample data. For the inference process, GMVAE approximates the true posterior distribution *p*(*z*,*y*|*x*) through variational inference *q*_*ϕ*_(*y*|*x*), similar to VAE. According to the mean-field approximation theory [24], *q*_*ϕ*_(*y*|*x*) can be further factorized as shown in Eq (5):

$$q_{\phi }(z,y|x)=q_{\phi }(z|x)q_{\phi }(y|x)$$ (5)

where $q_{\phi }(z|x)=N(z|\mu_{x},\sigma_{x}^{2})$ represents the latent distribution learned by the encoder network parameterized by *ϕ*, which gradually approximates the Gaussian distribution *p*(*z*|*y*) under the corresponding category during training. Similarly, *q*_*ϕ*_(*y*|*x*) is used to learn the categorical information from the input *x*, but this requires an additional neural network for fitting, thereby introducing more parameters to the model and increasing dependency on labeled data. To mitigate this, *p*(*y*|*z*) can be approximated as *q*_*ϕ*_(*y*|*x*) following the derivation process in VaDE [25], as shown in Eq (6):

$$q_{\phi }(y|x)=E_{q_{\phi }(z|x)}[p(y|z)]\approx \frac{1}{N}\sum_{n=1}^{N}\frac{p(z|y)p(y)}{\sum_{\hat{y}=1}^{K}p(z|\hat{y})p(\hat{y})}$$ (6)

This method leverages the properties of GMM and uses Bayesian inference to determine which Gaussian component’s latent distribution generates the data, thus identifying its category without introducing additional neural networks. Finally, Eq (6) is approximated through Monte Carlo estimation of the expectation *q*_*ϕ*_(*y*|*x*), where *N* represents the number of samples used for Monte Carlo estimation. In practice, the mean *μ*_*y*_ and variance $\sigma_{y}^{2}$ of each Gaussian component can be initialized using data with known categories. These parameters are then refined during the training process until they accurately reflect the categorical information. Unlabeled data are also utilized to update the parameters of the Gaussian components, further enhancing the model’s ability to learn categories.

Similar to VAE, GMVAE optimizes model parameters by maximizing the ELBO. Based on the aforementioned generative and inference processes, the log-likelihood function log *p*(*x*) can be defined as shown in Eq (7):

$$\log\;p(x)\geq E_{q_{\phi }(z|x)}[\log\frac{p_{\theta }(x,z,y)}{q_{\phi }(z,y|x)}]={ℒ}_{ELBO}$$ (7)

Under the semi-supervised approach, which uses both labeled and unlabeled data, ℒ_*ELBO*_ can be further expanded as shown in Eq (8):

$$\begin{array}{l} {ℒ}_{ELBO}=E_{q_{\phi }(z|x)}[\log p_{\theta }(x|z)]-\left\{ \begin{array}{l} {ℒ}_{\mathrm{KL}}^{\mathrm{unsup}},\;\mathrm{unsupervised}\\ {ℒ}_{\mathrm{KL}}^{\sup},\;\mathrm{supervised} \end{array}\right.\\ {ℒ}_{\mathrm{KL}}^{\mathrm{unsup}}=E_{q_{\phi }(y|x)}[\mathrm{KL}[q_{\phi }(z|x)\|p(z|y)]]+\mathrm{KL}[q_{\phi }(y|x)\|p(y)]\\ {ℒ}_{\mathrm{KL}}^{\sup}=\mathrm{KL}[q_{\phi }(z|x)\|p(z|y)] \end{array}$$ (8)

where $E_{q_{\phi }(z|x)}[\log\;p_{\theta }(x|z)]$ represents the probability of generating real data, which can be achieved by minimizing the cross-entropy reconstruction loss. ${ℒ}_{\mathrm{KL}}^{\mathrm{unsup}}$ represents the KL divergence loss under unsupervised learning, which requires minimizing the KL divergence between the latent distribution learned by the encoder and the latent distribution under the true category, as well as between the inferred posterior distribution of the category and the assumed uniform categorical distribution. ${ℒ}_{\mathrm{KL}}^{\sup}$ represents the KL divergence loss under supervised learning, where only the former KL divergence needs to be minimized.

### 3.2 Variance penalization and mutual information enhancement

When applying the GMVAE model to perform clustering inference on data, high data similarity and an insufficient number of Gaussian mixture components can hinder the learning of more complex distributions. In the latent space, each Gaussian component may struggle to separate during training, often resulting in components with closely aligned means and variances, and in extreme cases, multiple Gaussians may collapse into a single Gaussian. This phenomenon, known as mode collapse, is a significant challenge for GMVAE in distinguishing between different categories of data.

To alleviate the mode collapse issue in GMVAE, we further analyze the ELBO from Section 3.1 and identify the KL divergence regularization term as a primary contributor to this problem. Therefore, the KL divergence loss term ${\mathrm{KL}}_{z}=E_{q_{\phi }(y|x)}[\mathrm{KL}[q_{\phi }(z|x)\|p(z|y)]]$ in the latent distribution ${ℒ}_{\mathrm{KL}}^{\mathrm{unsup}}$ is re-expressed based on Eq (8) as:

$$\begin{array}{l} {\mathrm{KL}}_{z}=\sum_{\hat{y}=1}^{K}q_{\phi }(\hat{y}|x)E_{q_{\phi }(z|x)}\log\frac{q_{\phi }(\hat{y}|x)}{p(z|\hat{y})}\\ \;=E_{q_{\phi }(z|x)}\log\frac{q_{\phi }(\hat{y}|x)}{\Pi_{\hat{y}=1}^{K}p{\left(z|\hat{y}\right)}^{q_{\phi }(\hat{y}|x)}} \end{array}$$ (9)

where $E_{q_{\phi }(y|x)}=\sum_{\hat{y}=1}^{K}q_{\phi }(\hat{y}|x)=1$ denotes the Gaussian component under category $\hat{y}$, characterized by mean *μ*_*y*_ and variance $\sigma_{y}^{2}$. Assuming the variances of all Gaussian components are equal, the Gaussian distribution’s probability density function $p(z|\hat{y})$ can be expressed as:

$$p(z|\hat{y})=\frac{1}{\sqrt{2\pi \sigma_{y}^{2}}}\exp(-\frac{{\left(z-\mu_{\hat{y}}\right)}^{2}}{2\sigma_{y}^{2}})$$ (10)

Let $\Pi_{\hat{y}=1}^{K}p{\left(z|\hat{y}\right)}^{q_{\phi }(\hat{y}|x)}$ represent $f(x,\hat{y},z)$, and combining with Eq (10), it can be represented as:

$$\begin{array}{l} f(x,\hat{y},z)=\frac{1}{\sqrt{2\pi \sigma_{y}^{2}}}\exp(-\frac{1}{2\sigma_{y}^{2}}\sum_{\hat{y}=1}^{K}q_{\phi }(y|x){\left(z-\mu_{\hat{y}}\right)}^{2})\\ \;=\frac{1}{\sqrt{2\pi \sigma_{y}^{2}}}\exp[-\frac{1}{2\sigma_{y}^{2}}{\left(z-E_{q_{\phi }(y|x)}\mu_{y}\right)}^{2}]\\ \;\cdot \exp[-\frac{1}{2\sigma_{y}^{2}}(E_{q_{\phi }(y|x)}\mu_{y}^{2}-E_{q_{\phi }(y|x)}^{2}\mu_{y})] \end{array}$$ (11)

The first factor of the product can be regarded as the average Gaussian component across all categories *p*(*z*|*y*), while the second factor represents the variance of the means of all Gaussian components, which measures the dispersion of these components. Combining Eqs (9) and (11), KL_*z*_ can be transformed, as shown in Eq (12):

$${\mathrm{KL}}_{z}=\mathrm{KL}[q_{\phi }(z|x)\|p(z|y)]+\frac{1}{2\sigma_{y}^{2}}(E_{q_{\phi }(y|x)}\mu_{y}^{2}-E_{q_{\phi }(y|x)}^{2}\mu_{y})$$ (12)

When maximizing GMVAE’s ELBO during training, the variance term factor in KL_*z*_ also gets minimized, causing the variance of the means of Gaussian components to shrink, leading to tighter connections between Gaussian components in the latent space and impairing the model’s ability to correctly distinguish between categories, thereby causing mode collapse. To reduce this contraction trend, we introduce a penalization hyperparameter *α*, *α*∈[0, 1] before the variance term in KL_*z*_:

In addition to improving the separability of Gaussian components to enhance clustering accuracy, establishing a strong connection between the input data and category information is essential. However, research has shown that VAE’s ELBO has an information suppression term that weakens the relationship between input data and latent variables [26]. This bottleneck also exists between the input data and category information in GMVAE. During model training, the average ELBO of a batch of data is typically computed for gradient updates. Therefore, combined with Eq (12), ℒ_*ELBO*_ can be expanded as shown in Eq (13):

$${ℒ}_{ELBO}=E_{x}[E_{q_{\phi }(z|x)}[\log\;p_{\theta }(x|z)]]-{\mathrm{KL}}_{z}-\mathrm{KL}[q_{\phi }(y|x)\|p(y)]$$ (13)

According to the definition of mutual information concerning KL divergence [27], the mutual information MI(*y*,*x*) between input data *x* and category information *y* can be expressed as shown in Eq (14):

$$\mathrm{MI}(y,x)=E_{x}[\mathrm{KL}[q_{\phi }(y|x)\|p(y)]]-\mathrm{KL}[q_{\phi }(y|x)\|p(y)]$$ (14)

Therefore, ℒ_*ELBO*_ can be rewritten as shown in Eq (15):

$${ℒ}_{ELBO}=E_{x}[E_{q_{\phi }(z|x)}[\log\;p_{\theta }(x|z)]-{\mathrm{KL}}_{z}]-\mathrm{MI}(y,x)-\mathrm{KL}[q_{\phi }(y)\|p(y)]$$ (15)

This shows that during training, the mutual information between input data and category information is minimized, reducing the dependence between these two random variables and diminishing clustering accuracy. Therefore, we propose a joint optimization of ELBO and mutual information, defined as:

$${ℒ}_{ELBO}+\mathrm{MI}(y,x)=E_{x}[E_{q_{\phi }(z|x)}[\log\;p_{\theta }(x|z)]-{\mathrm{KL}}_{z}]-\mathrm{KL}[q_{\phi }(y|x)\|p(y)]$$ (16)

This method still allows for optimizing the learning of the categorical distribution through $\mathrm{KL}[q_{\phi }(y|x)\|p(y)]$, where $q_{\phi }(y)=E_{x}[q_{\phi }(y|x)]$ represents the discrete marginal probability distribution of *q*_*ϕ*_(*y*|*x*), which can be averaged over data points within a batch, reducing the difference between the categorical distribution across all data points and the prior distribution.

Finally, combining variance penalization and mutual information optimization yields the unsupervised KL divergence loss for GMVAE, as shown in Eq (17):

$${ℒ}_{\mathrm{KL}}^{\mathrm{unsup}}=\mathrm{KL}[q_{\phi }(z|x)\|p(z|y)]+\frac{\alpha }{2\sigma_{y}^{2}}(E_{q_{\phi }(y|x)}\mu_{y}^{2}-E_{q_{\phi }(y|x)}^{2}\mu_{y})+\mathrm{KL}[q_{\phi }(y|x)\|p(y)]$$ (17)

These two enhancement methods ensure that data from different categories are better separated in the latent space, while data from the same category are more compactly grouped.

### 3.3 CDGMVAE objective function

In supervised learning, the loss function should include not only the corresponding KL divergence loss but also a classification cross-entropy loss function to guide the learning of categories. The classification loss is defined as shown in Eq (18):

$${ℒ}_{label}=-\sum_{c=1}^{K}y_{c}\;\log q_{\phi }(y_{c}|x)$$ (18)

where *K* is the total number of categories, *y*_*c*_ represents the true label indicating whether the sample belongs to the *c*_th_ category, and *q*_*ϕ*_(*y*_*c*_|*x*) is the predicted probability for the *c*_th_ category.

Additionally, by integrating the disentanglement loss ℒ_*Dis*_ discussed in Section 2, we can derive the final objective loss function for the CDGMVAE model. Given the latent variable *z*_*i*_ of the music feature *f* and the category variable *y*_*i*_, the objective loss function can be described by Eq (19):

$$\begin{array}{c} {ℒ}_{\mathrm{CDGMVAE}}=E_{q_{\phi_{r}}(z_{r}|x)q_{\phi_{k}}(z_{k}|x)}[\log\;p_{\theta }(x|z_{r},z_{k})]+{ℒ}_{Dis}\\ -\sum_{i}|\begin{array}{l} {ℒ}_{\mathrm{KL}}^{\mathrm{unsup},i},\;\;\mathrm{unsupervised}\\ {ℒ}_{\mathrm{KL}}^{\sup,i}-{ℒ}_{\mathrm{label}}^{i},\;\mathrm{supervised} \end{array} \end{array}$$ (19)

### 3.4 Encoder and decoder

**Fig 4:**
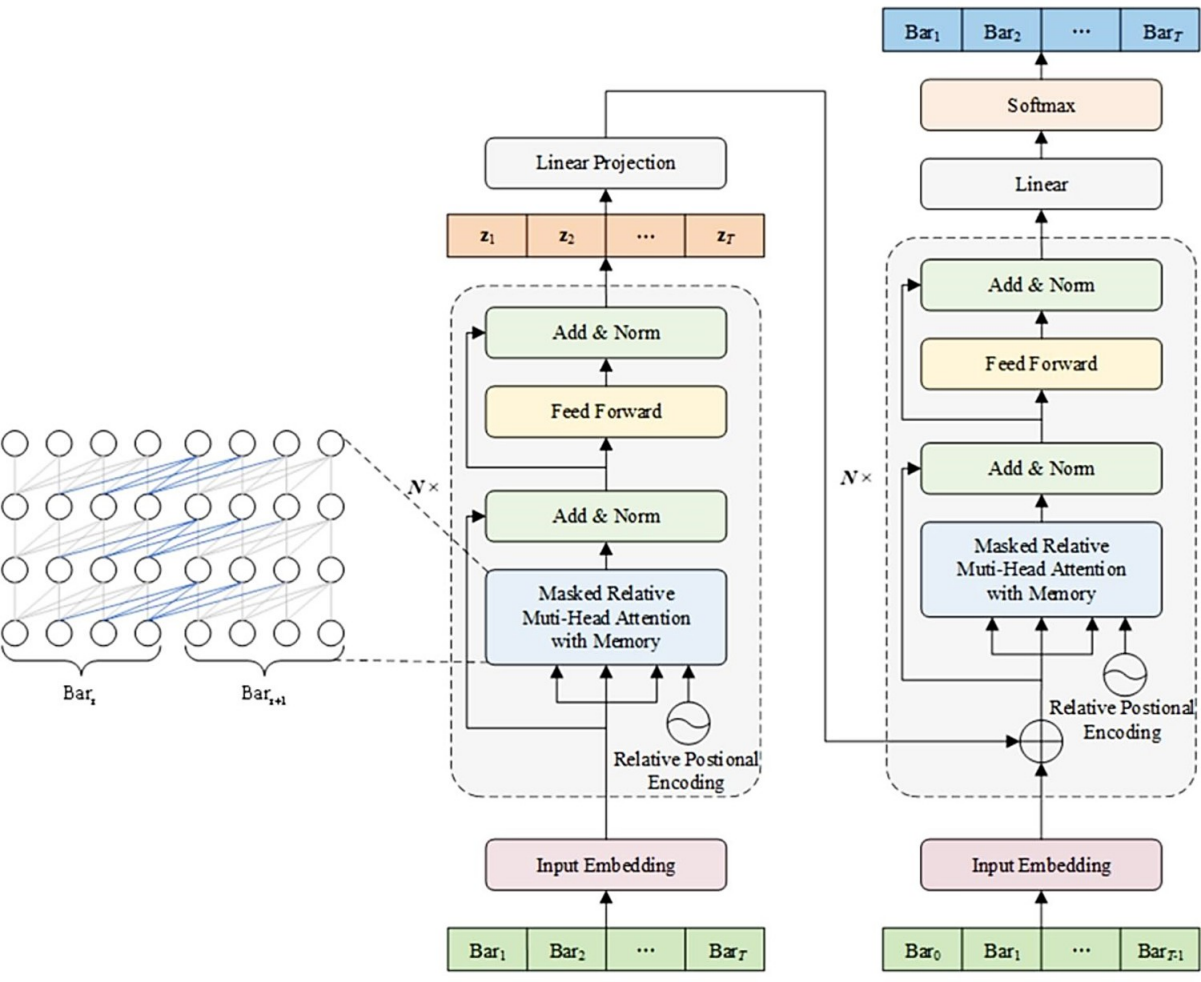
Structure of the CDGMVAE model based on Transformer-XL.

Traditional VAE models and their variants often use RNNs to construct the encoder and decoder. However, due to RNNs’ limited modeling capabilities for long sequence data, the Transformer architecture [28] has also been considered in conjunction with VAE models for music generation, as seen in models like Transformer-VAE [29] and MuseMorphose [30]. However, the Transformer’s input sequence length is still constrained by memory, requiring self-attention calculations over the entire sequence each time. To address this issue, the CDGMVAE model adopts the Transformer-XL network structure, as shown in **Fig 4**. Transformer-XL leverages segment-level recurrence and relative positional encoding to learn the contextual structure across more extended musical bars.

In this model, the music sequence is divided into multiple segments of equal length, denoted as *x* = [*Bar*_1_,*Bar*_2_,⋯,*Bar*_*T*_], where *T* represents the number of bars in each segment. Let $h_{\tau -1}^{n-1}\in {\mathbb{R}}^{l\times d}$ be the hidden state of the (*τ*-1)_th_ bar in the n-1 layer of self-attention, where *L* is the sequence length of each bar, and *d* is the dimension of the hidden state. When calculating the hidden state $h_{\tau }^{n}$ of the *τ*_th_ bar in the n_th_ layer, the model considers reusing the hidden state from the previous bar. The calculation process is shown in Eq (20):

$$\begin{array}{l} {\tilde{h}}_{\tau }^{n-1}=Concat[SG(h_{\tau -1}^{n-1})\circ h_{\tau }^{n-1}]\\ Q_{\tau }^{n},K_{\tau }^{n},V_{\tau }^{n}=h_{\tau }^{n-1}W_{Q}^{T},{\tilde{h}}_{\tau }^{n-1}W_{K}^{T},{\tilde{h}}_{\tau }^{n-1}W_{V}^{T}\\ h_{\tau }^{n}=Transformer-layer(Q_{\tau }^{n},K_{\tau }^{n},V_{\tau }^{n}) \end{array}$$ (20)

where *SG*(⋅) indicates that the gradient of $h_{\tau -1}^{n-1}$ will not be updated when calculating the next bar, *Concat*(⋅) represents the concatenation of the two hidden state matrices along the bar length, and *W*_*Q*,*K*,*V*_ are the learnable model parameters. Unlike the Transformer, during the attention score calculation, the key-value matrices $K_{\tau }^{n}$ and $V_{\tau }^{n}$ depend on the hidden state $h_{\tau -1}^{n-1}$ of the previous bar in the *n*-1 layer (as shown by the blue lines in **Fig 4**) and the hidden state $h_{\tau }^{n-1}$ of the current bar in the same layer (as shown by the gray lines in **Fig 4**). This method allows the model to cache only the hidden state of the previous bar in memory, establishing a recurrent connection between segments and extending contextual information across all bars.

Transformer models utilize positional encoding to learn the order and positional relationships within an input sequence. However, this positional encoding is absolute, meaning that the encoding information for the same position across different bars is identical, potentially causing the model to misinterpret the sequence. To address this issue, Transformer-XL introduces a relative positional encoding mechanism, incorporating a parameter-free sinusoidal encoding matrix *R*∈ℝ^*L*×*d*^. Each row of this matrix represents the relative distance encoding between two positions. When computing hidden states, this relative distance can be dynamically injected into the attention scores, allowing the model to better distinguish between different positions.

When integrating Transformer-XL with the GMVAE model, the goal is to learn the latent variable representations of the data from the hidden states output by the encoder. According to GMVAE’s definition of latent distribution *q*(*z*|*x*), the model first learns the mean and variance of this distribution, then samples the latent variables. This process can be described by Eq (21):

$$\begin{array}{l} h_{\tau }^{pool}=Avgpool([h_{\tau ,1}^{N},h_{\tau ,2}^{N},\cdots ,h_{\tau ,L}^{N}])\\ \mu =h^{pool}W_{\mu },\log\sigma^{2}=h^{pool}W_{\sigma }\\ z=\mu +\varepsilon ⊙\sigma \varepsilon ∼N(0,I) \end{array}$$ (21)

where $h_{\tau }^{pool}\in {\mathbb{R}}^{d}$ represents the overall hidden state of the τ_th_ bar, obtained by average pooling across all positions’ hidden states. *μ*,*σ*∈ℝ^*T*×*l*^ denotes the mean and standard deviation vectors of the latent distribution learned from the hidden states, with *l* representing the set dimension of the latent variable, and *W*_*μ*_,*W*_*σ*_∈ℝ^*d*×*l*^ is the parameter matrix. *z*∈ℝ^*T*×*l*^ is the sampled latent variable from the learned distribution using the reparameterization trick. During the decoding and generation phase, the latent variables are transformed through a linear layer to match the unified model dimension. They are then added to the hidden states output from the previous layer before being input into the subsequent self-attention layer. This method ensures that the latent variable information is effectively utilized to generate the final music sequence.

## 4. Experimental results and analysis

### 4.1 MIDI music dataset

In this study, the publicly available Lakh Pianoroll dataset (LPD) [31] is utilized, comprising 174,154 multi-track MIDI music sequences in piano roll format. We selected the LPD-5-full version, excluding sequences with duplicate content, those lacking piano tracks, and those with a total duration of less than one minute. This process resulted in a dataset of 91,969 sequences, which is split into training, validation, and test sets in an 8:1:1 ratio. Additionally, the dataset is matched with the Million Song Dataset (MSD) [32], enabling the retrieval of emotional metadata for 23,967 music sequences using the metadata provided by MSD and the API services from the Spotify music community (developer.spotify.com). These sequences were annotated in the Russell emotional space with Arousal and Valence values, which were then quantified into two emotional dimensions (high or low) and labeled as A0, A1, V0, and V1, where 0 represents low and 1 represents high. By combining these labels, four corresponding emotional categories were defined: happiness, tension, sadness, and calmness.

### 4.2 Music data representation

To model MIDI music using the CDGMVAE framework, an appropriate data representation is required. Python libraries such as Pretty_midi and music21 were employed to parse the structural information of MIDI music, and the REMI [33] format is used to encode the music into a sequence of tokenized events. Each event type represents a temporal change in the musical sequence, such as a Bar event indicating the beginning of a measure or a Beat event marking the start of a beat, with corresponding Tempo, Pitch, Velocity, and Duration tokens. **Table 1** outlines the specific meaning of each event type. For the LPD dataset, each music piece can contain up to five tracks. Pitch, Velocity, and Duration are track-specific events, thus denoted as Pitch-[Track], Velocity-[Track], and Duration-[Track] to represent note events on a particular track. An EOS token is appended to the end of each sequence to signify its conclusion, and a PAD token is used for sequence padding, resulting in a total of 923 tokens. During data loading, we randomly selected continuous measures, setting the number of measures *T* to 20 and the maximum sequence length per measure *L* to 128.

**Table 1:** MIDI music event information.

Event name	Event description	Token count
Bar	Start of a measure	1
Beat	Start of a sub-beat within a measure, with each measure divided into 16 beats	16
Tempo	Tempo of the music at a given time, ranging from 32 to 224 bpm with a step size of 3 bpm	64
Pitch	Pitch of a note, ranging from 21 to 108	88×5
Velocity	Intensity of a note, ranging from 1 to 127 with a step size of 2	64×5
Duration	Duration of a note, quantized as the length in beats, 1≤n≤16	16×5
EOS	End of sequence	1
PAD	Padding token	1

For disentangled feature learning, rhythm and key features were selected as the musical attributes linked to emotions. The rhythm feature is represented as a 16×*T* dimensional vector, defined by the number of simultaneous notes played in each beat. Higher note frequencies within a time span indicate more intense rhythm, reflecting a higher arousal level, whereas lower frequencies correspond to a more moderate and calm rhythm. The key feature is represented as a T-dimensional vector, defined by the possible key signatures within each measure. The keys are categorized into 12 major (e.g., C major) and 12 minor scales (e.g., c minor), with major keys typically conveying positive emotions and minor keys conveying negative emotions, thus corresponding to the valence dimension of emotions. By leveraging these two features within the CDGMVAE model, control over the emotional content of the generated music can be achieved.

### 4.3 Experimental setup and parameter settings

The CDGMVAE model is developed using the PyTorch deep learning framework and trained on a server equipped with four NVIDIA Tesla P40 GPUs. For the CDGMVAE network structure, the number of layers in both the Transformer-XL encoder and decoder is set to 12. Each self-attention mechanism layer used 8 heads, with hidden layers and latent variable dimensions set to 512 and 128, respectively. To train the Gaussian Mixture Model (GMM) on the Arousal and Valence emotional dimensions, the number of Gaussian components for each dimension is set to 2, representing high and low dimensions. The Xavier initialization method [34] is employed to initialize each Gaussian component’s mean vector, with initial standard deviation vectors set to a constant value of e^-2^. During training, a batch size of 16 is used, and the Adam optimizer is employed for parameter updates, with dynamic learning rate adjustments to facilitate model convergence. For the first 10,000 iterations, a linear warm-up is applied to increase the learning rate from 0 to 1×e^−4^. Subsequently, in the next 220,000 iterations, a cosine annealing strategy is used to gradually decrease the learning rate to 5×e^−6^. The model achieved convergence after 50 training epochs.

### 4.4 Experimental analysis

#### 4.4.1 Semi-supervised generation performance

This section evaluates the semi-supervised generation performance of the CDGMVAE model by comparing it with three fully supervised emotional music generation models and three semi-supervised models. The supervised models include the Transformer-based EMOPIA [35], the GRU-based EmotionBox [36], which links music features with emotions, and the Conditional Variational Autoencoder (CVAE) model [37] that conditions on emotional labels. The semi-supervised models assessed are SSVAE [38], CCVAE [39], and FaderNets [40]. To ensure a fair comparison, all semi-supervised models utilized Transformer-XL for the core network structure, with the exception of SSVAE, which incorporated feature disentanglement mechanisms. Additionally, a music emotion classification model provided by EMOPIA is trained on labeled data from LPD, using 8-bar music sequences as input. After 50 training epochs, the classification performance achieved accuracy comparable to that reported in the original paper. Furthermore, a trained classifier is used to predict the emotional dimensions of Arousal and Valence for all generated music samples. The trained classifier adopts three-layer fully connected layer with parameters of 128-50-10-1. Performance is evaluated using accuracy (Acc), precision (Pre), recall (Recall), and F1 score (F1).

The generation performance of various models under both fully supervised and semi-supervised methods is first compared. Results are shown in Tables 2 and 3, corresponding to the Arousal and Valence emotional dimensions, respectively. Tables 2 and 3 indicate that, despite all models being trained in a supervised manner, EMOPIA, EmotionBox, and CVAE generally perform worse than other generation models. This can be attributed to the limited contextual learning capabilities of GRU and Transformer compared to Transformer-XL, and the fact that some models use emotional labels directly, lacking representational power compared to feature disentanglement methods. The results also reveal that models trained with semi-supervised methods, incorporating additional unlabeled data, significantly outperform those trained fully supervised across all classification metrics. This demonstrates that VAE-based generation models and their variants effectively utilize unlabeled data to learn broader distribution information, with labeled data providing constraints on categorical information. Overall, CDGMVAE and FaderNets exhibit better generation performance compared to the other two semi-supervised models. This is because the GMVAE model incorporates label information and uses Gaussian mixture distributions to separate the latent space into multiple discrete regions, effectively learning diverse data distributions. In contrast, other semi-supervised VAE models rely on additional neural networks to learn categorical information, which may overly depend on labeled data, limiting the classifier’s ability to fully model data distributions. Moreover, the CDGMVAE’s enhanced variance and mutual information terms lead to higher classification accuracy compared to FaderNets. This indicates that CDGMVAE better learns latent variable representations for each Gaussian component and accurately distinguishes between different emotional categories, a point further explored in subsequent sections.

**Table 2:** Performance comparison of supervised and semi-supervised methods in Arousal dimension.

Models	Supervised				Semi-Supervised			
	Acc/%	Pre/%	Rec/%	F1/%	Acc/%	Pre/%	Rec/%	F1/%
EMOPIA	61.55	58.69	61.52	60.07	-			
EmotionBox	60.06	57.35	58.71	58.02	-			
CVAE	58.63	55.99	56.11	56.05	-			
SSVAE	61.28	58.84	58.73	58.78	73.66	71.94	72.11	72.02
CCVAE	63.74	61.49	61.21	61.35	76.24	75.58	73.07	74.30
FaderNets	68.37	65.21	70.17	67.60	78.65	76.54	78.73	77.62
CDGMVAE	**70.52**	**66.92**	**73.77**	**70.18**	**82.54**	**79.56**	**84.60**	**82.00**

**Table 3:** Performance comparison of supervised and semi-supervised methods in Valence dimension.

Models	Supervised				Semi-Supervised			
	Acc/%	Pre/%	Rec/%	F1/%	Acc/%	Pre/%	Rec/%	F1/%
EMOPIA	57.84	59.81	57.82	58.80	-			
EmotionBox	57.18	59.06	57.66	58.35	-			
CVAE	55.27	59.96	57.34	57.15	-			
SSVAE	58.12	59.96	59.70	59.32	67.15	68.00	69.61	68.80
CCVAE	60.03	61.67	63.43	62.28	69.30	72.88	65.28	68.87
FaderNets	63.45	64.94	64.64	64.79	71.63	72.44	73.38	72.91
CDGMVAE	**66.63**	**68.93**	**65.28**	**67.05**	**75.87**	**77.37**	**75.78**	**76.57**

**Fig 5:**
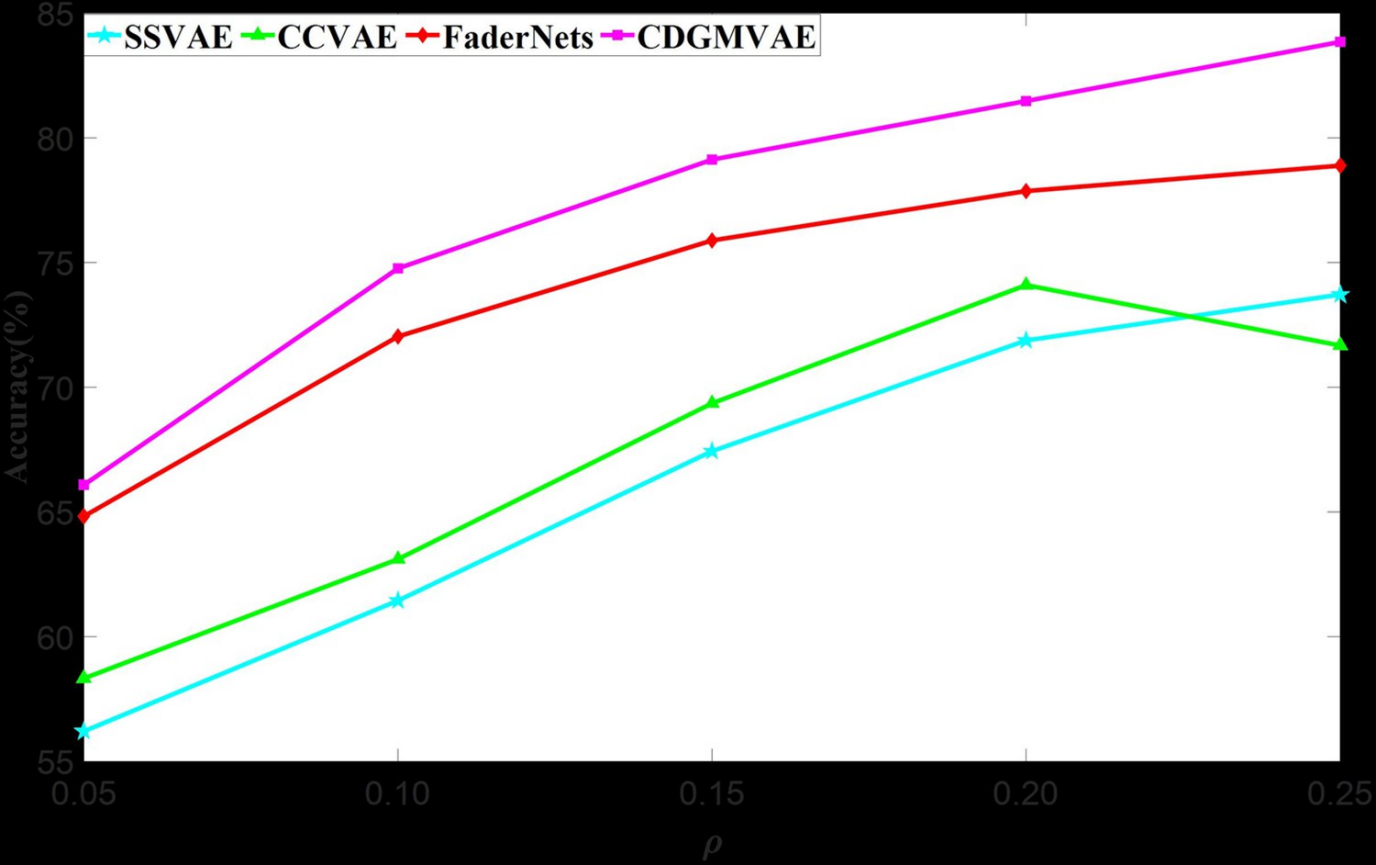
Accuracy of generated music in the Arousal dimension.

**Fig 6:**
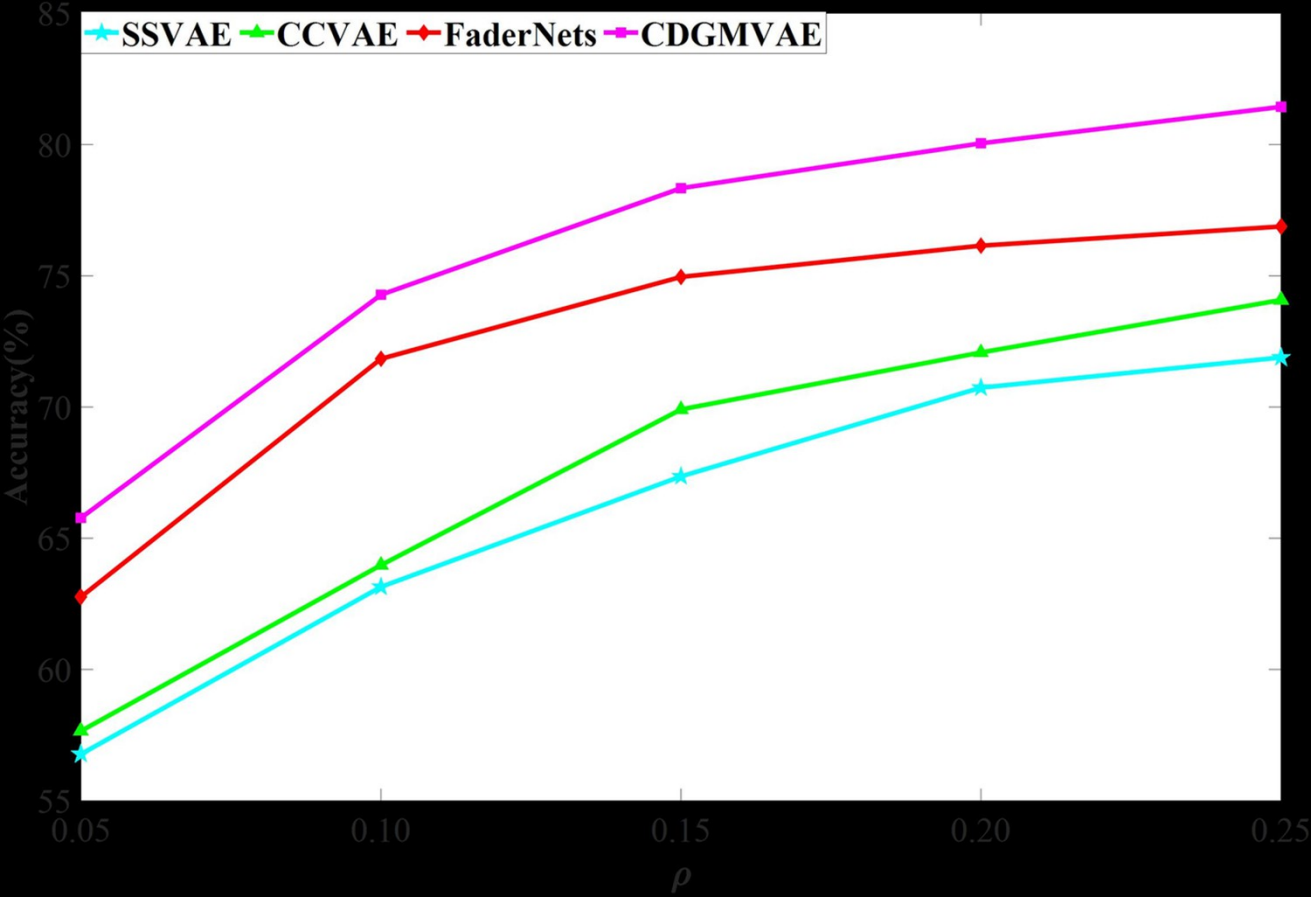
Accuracy of generated music in the valence dimension.

To further illustrate the effectiveness of semi-supervised learning, we tested the accuracy of generated music across different amounts of labeled data. Results are presented in Figs 5 and 6, depicting accuracy in the Arousal and Valence emotional dimensions under various supervision rates. Here, *ρ* = *M*/(*M*+*N*), where *M* denotes the number of labeled data samples and *N* represents the total amount of unlabeled data, with 0<α≤0.26. Figs 5 and 6 show that CDGMVAE and FaderNets achieve higher accuracy even with a supervision rate of 0.05. This suggests that GMVAE can learn extensive data distribution across emotional categories with only a small amount of labeled data. Additionally, due to the optimization of variance and mutual information in CDGMVAE, it consistently outperforms FaderNets in accuracy under all supervision conditions. This confirms that the absence of diversity among Gaussian components and insufficient linkage between data and emotional categories can lead to errors in category inference, thereby reducing the emotional quality of generated music. For SSVAE and CCVAE, results indicate that their accuracy is significantly lower than that of GMVAE-based models across all supervision rates, due to their limited ability to learn meaningful latent representations for specific emotions from the classifier. Although SSVAE and CCVAE show a slightly greater increase in accuracy compared to GMVAE models and have considerable potential for improvement, this also highlights their over-reliance on labeled data for supervision signals. Among them, CCVAE exhibits better performance than SSVAE, likely due to its feature disentanglement approach, allowing the classifier to learn emotional categories from rhythm and key features, thereby incorporating the necessary emotional information into latent variables. In contrast, SSVAE directly learns emotional category information from the raw input, which can be less effective in capturing subtle emotional details during the decoding process.

In summary, the music generated by all models is more easily distinguishable in the Arousal dimension compared to the Valence dimension. This is attributed to the more universal and direct nature of rhythm features, which can effectively capture latent representations from raw input data. In contrast, key features require complex music theory inference, which the models may not adequately possess. The experiments also demonstrate that rhythm features are more closely related to the Arousal dimension and are better at influencing the activity level of the music. The Valence dimension involves emotional polarity, which cannot be fully represented by key features alone. This finding aligns with conclusions drawn from the EmotionBox model.

#### 4.4.2 Variance penalty and mutual information enhancement performance analysis

**Fig 7:**
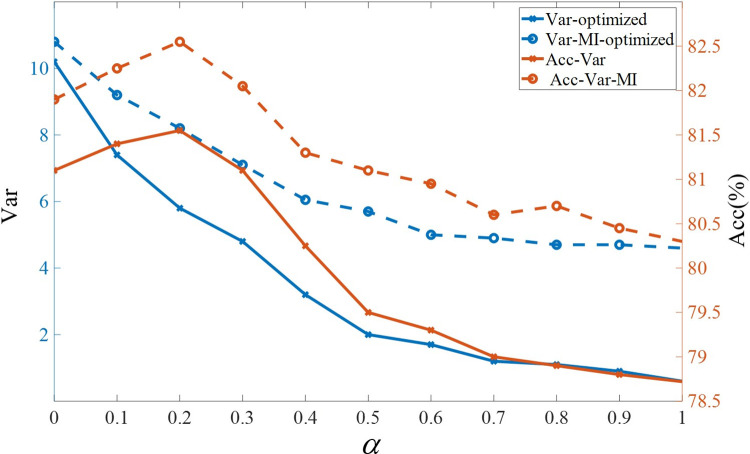
Impact of variance penalty and mutual information enhancement on Arousal dimension.

**Fig 8:**
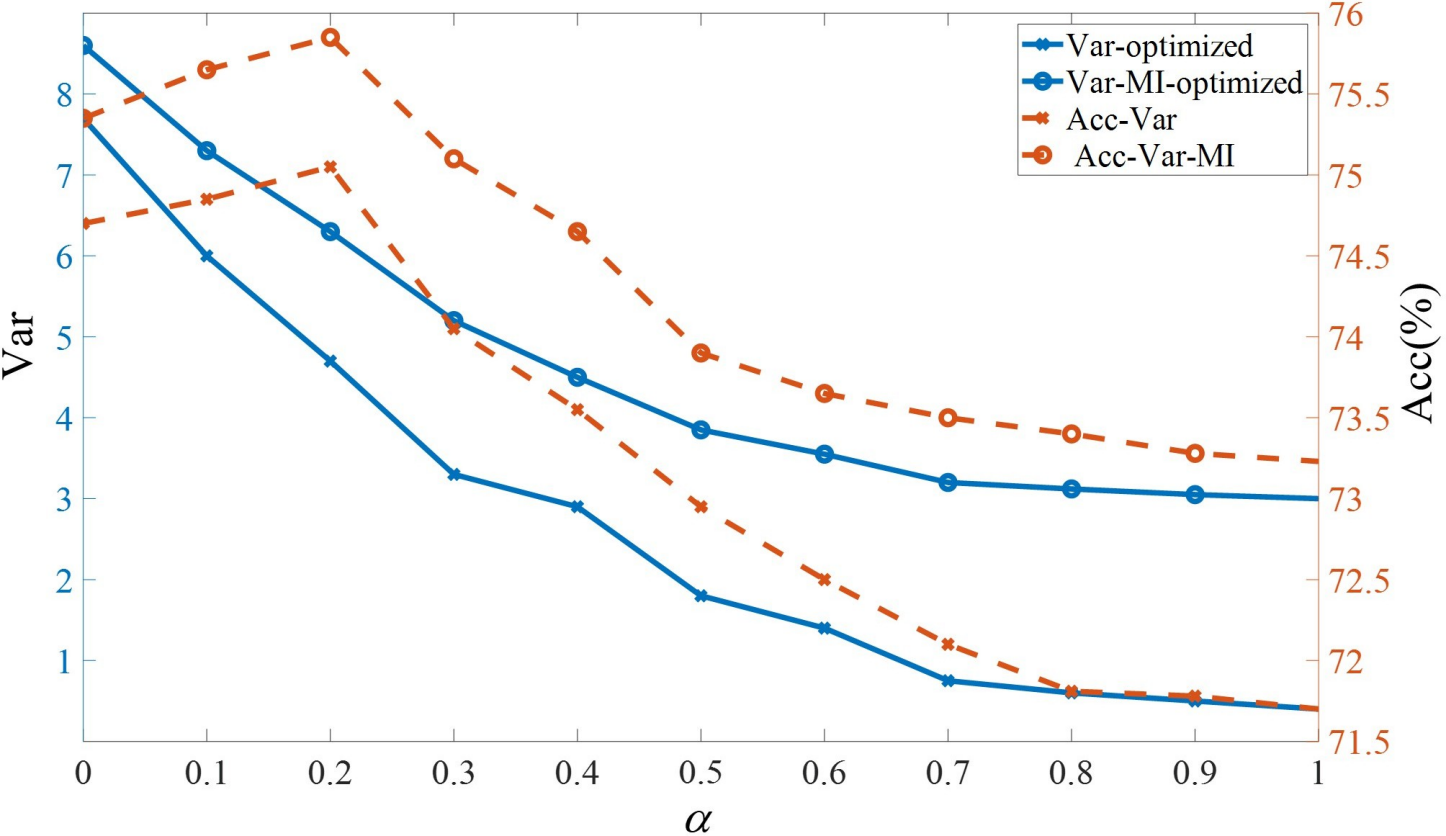
Impact of variance penalty and mutual information enhancement on Valence dimension.

**Fig 9:**
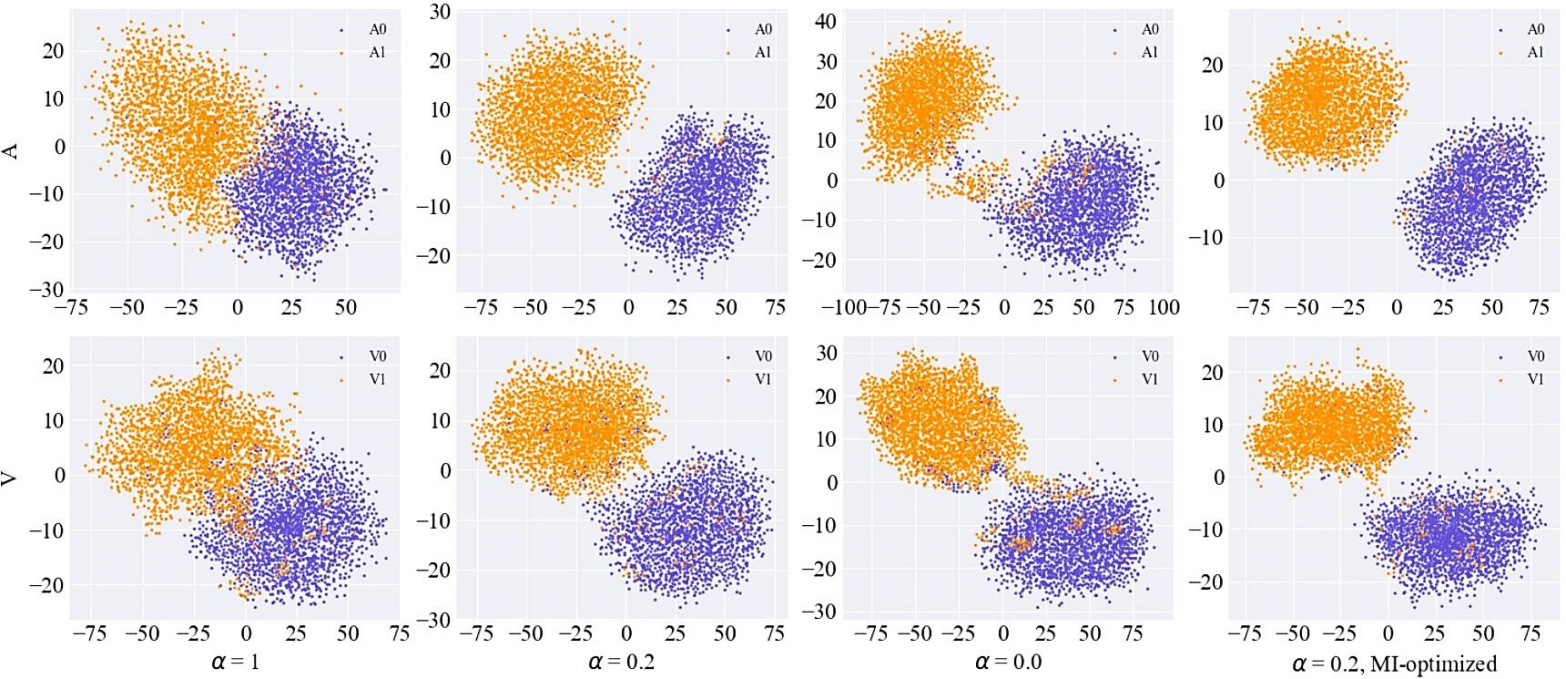
Visualization of latent space under the influence of variance penalty and mutual information enhancement.

This subsection discusses how the variance and mutual information penalty terms in the GMVAE evidence lower bound affect the latent space and demonstrates that appropriate correction of these biases enables the model to perform reasonable emotional category inference and generation for music data. Initially, we compared the trends in variance of Gaussian components and their impact on emotional accuracy of generated music under varying penalty weights (i.e., *α* values). The experimental results are illustrated by the blue lines in Figs 7 and 8. To provide a clearer view of the variations among Gaussian components, we used the T-SNE dimensionality reduction algorithm to project high-dimensional latent variables into a two-dimensional space for visualization. The results are shown in Fig 9.

As analyzed in Section 3.2, lower values of *α* reduce the influence of the variance term on the overall objective function, resulting in higher variance and classification accuracy. Correspondingly, the distances between Gaussian components in the latent space visualization increase. This enhances the model’s ability to generate target emotions in the music effectively. However, when *α*≤0.2, while the distances between Gaussian components reach their maximum, the accuracy of emotional classification decreases. The latent space visualization reveals that unnecessary cluster centers form between the two Gaussian components, leading to more frequent misclassifications, with data from one class being assigned to another cluster. This issue arises because excessive penalty strength can cause overfitting, preventing the model from capturing the overall data distribution and resulting in information loss. Therefore, based on experimental results, a *α* value of 0.2 is chosen to optimize model performance. Furthermore, the red lines in Figs 7 and 8 demonstrate the performance changes after enhancing mutual information on top of the variance penalty. With the same weight control, optimizing mutual information results in higher variance and accuracy, and at lower *α* values, mutual information enhancement compensates for insufficient variance penalty. **Fig 9** shows that after enhancing mutual information, the distances between different clusters become more pronounced, and within-cluster distances become more compact.

#### 4.4.3 Performance analysis of feature disentanglement

For emotion-based music generation, we incorporated a disentanglement mechanism into the CDGMVAE model, learning the emotional representations of rhythm and key features in the Arousal and Valence dimensions, respectively. This section evaluates the impact of changing feature representations on the generated music. Ideally, the two learned feature representations should be independent. For instance, altering the rhythm feature representation should change the rhythm pattern of the generated music, while the key feature remains unchanged. To validate the effectiveness of disentanglement learning, several comparison models were constructed and evaluated under the same experimental settings:

1. EC-VAE [41]: This model uses a bidirectional GRU-based VAE to disentangle pitch and rhythm representations from a single encoder’s overall latent variable, and then reconstructs rhythm features and the original input using rhythm and global decoders, respectively.
2. GAN-CVAE [42]: This model defines a latent space where the latent representation is independent of feature values. It learns a generalized latent variable representation devoid of any feature information through adversarial mechanisms, with each controllable feature divided into different categories. These categories are used as conditions to generate music with specific features, and the model is also built using GRU.
3. MuseMorphose [43]: Similar to GAN-CVAE, this model uses Transformer for the main network, focusing more on overall dependency information. It also explores different ways to inject latent variables and feature information into the decoder, effectively utilizing known conditional information.
4. Vanilla CDGMVAE: A simplified version of our model, inspired by the design in [44], which includes only the GMVAE evidence lower bound without additional loss function constraints.
5. CDLSTM-GMVAE: A variant of our model using bidirectional long short-term memory (BiLSTM) networks to construct the GMVAE model.

For feature controllability evaluation, we performed experiments where pairs of samples from the test set were input into the model’s encoder. Each pair of samples, denoted as A and B, had their latent variable representations for one feature swapped while keeping the other feature unchanged. The generated music is then compared with the original samples in terms of feature similarity. The similarity is assessed by calculating the cosine similarity between the generated samples and A, B after feature swapping, as defined in Eq (22).

sim(a,b)=〈a,b〉/‖a‖‖b‖ (22)

where *a* and *b* denote the feature vectors of generated and original samples, respectively, while 〈⋅, ⋅〉 represents the dot product, and ‖⋅‖ signifies the norm of the feature vector.

**Fig 10:**
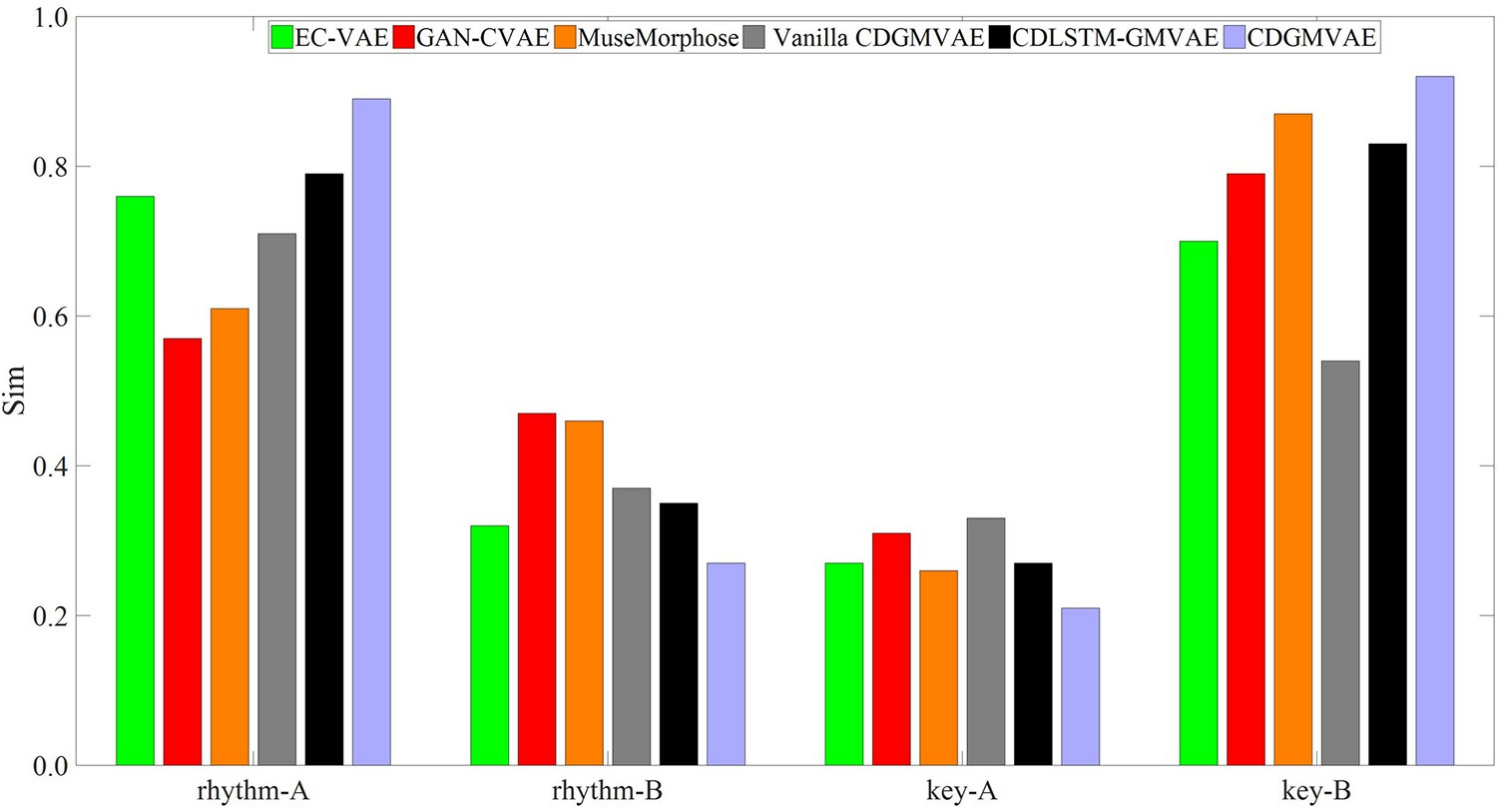
Feature similarity between generated samples and original samples after exchanging rhythmic feature representations.

**Fig 11:**
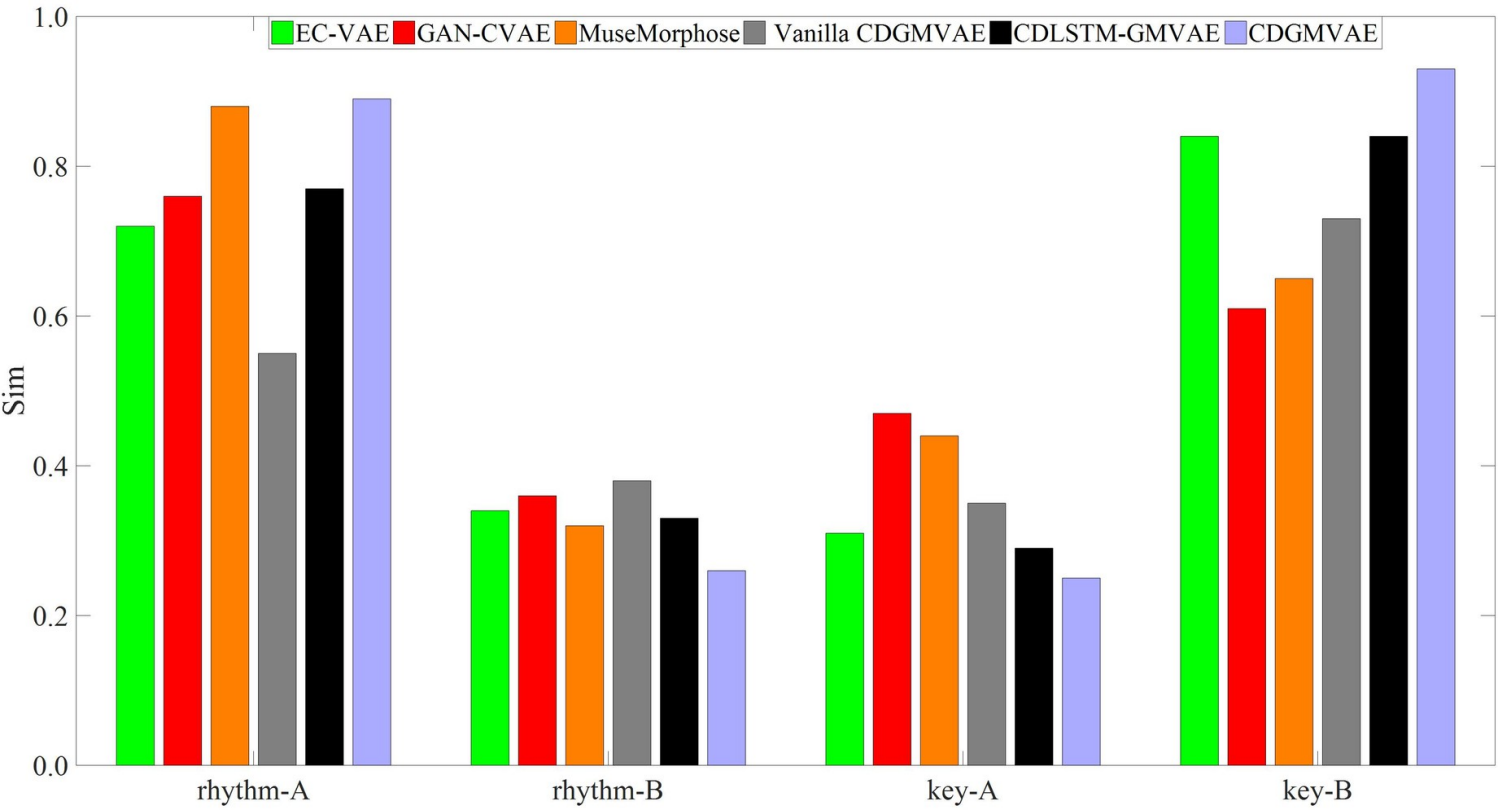
Feature similarity between generated samples and original samples after exchanging melodic feature representations.

Figs 10 and 11 display bar charts illustrating the variation in feature similarity between the generated samples and original samples A and B after exchanging rhythmic and melodic features, respectively. It is evident from the figures that the CDLSTM-GMVAE and CDGMVAE models effectively disentangle and separate features. Consequently, altering the latent variables of one feature does not significantly impact another feature. When substituting the latent variables of sample A with those of sample B, the generated music closely mimics the rhythmic and melodic patterns of sample B, resulting in higher feature similarity values. Conversely, when proper feature constraints are absent, such as in the Vanilla CDGMVAE and EC-VAE models, although the similarity between features of modified sample A and sample B is high, changes in one feature affect the other, leading to a reduced similarity with the original sample A. This indicates that the rhythmic and melodic feature representations have not been effectively disentangled.

Additionally, for the GAN-CVAE and MuseMorphose models, the effect of feature control is less pronounced compared to other models. The feature similarity with original sample A remains high, while similarity with substituted sample B is lower. This suggests that defining unrelated latent spaces for features and segmenting musical features into categorical intervals, while using generalized latent variables and categorical feature labels for music generation control, does not yield satisfactory results. The models tend to learn general data distributions and may overlook subtle feature information. In contrast, the latent space in CDGMVAE directly reflects semantic information related to features. Using latent variables representing specific features provides more detailed information, allowing the generation model to control the output more precisely. Thus, for emotion-driven music generation models controlled by features, it is crucial not only to have effective feature representations but also to ensure that these representations are independent and do not interfere with one another.

Through the above experimental analysis, the proposed emotion-driven music generation model, CDGMVAE, successfully disentangles rhythmic and melodic feature representations related to emotions from the original music sequences. It effectively manipulates these feature representations to achieve desired emotional variations in the Arousal and Valence dimensions. (More experiment details can be seen in the supplementary material [S1 Text])

#### 4.4.4 Interpolative emotion transformation

In the previous sections, the disentangled control of rhythmic and melodic features for music emotion transformation is assessed by exchanging features between two pieces of music. To fully leverage the discrete latent space of CDGMVAE, we employed a feature disentanglement mechanism combined with interpolation methods to enable mutual transformation of musical emotions by mapping the latent variables of the current music to the latent space of the target emotion. When transforming between high and low dimensions of a specific emotional dimension, the difference between the Gaussian component means *μ*_*i*,target_ in the target emotion space and the Gaussian component means *μ*_*i*,source_ in the current emotion space is first computed. This difference is then added to the latent variable *z*_*i*,source_ of the current emotion to obtain the latent variable *z*_*i*,target_ corresponding to the target emotion. Finally, this latent variable is input into the CDGMVAE decoder to generate a new sample. The entire process is described by Eq (23):

$$z_{i,\mathrm{target}}=z_{i,\mathrm{source}}+\lambda \cdot (\mu_{i,\mathrm{target}}-\mu_{i,\mathrm{source}})$$ (23)

where *i* denotes the emotion dimension to be transformed, and the parameter *λ*∈[0,1] controls the degree of closeness between the current and target emotions. For clearer visualization of emotion changes in this experiment, *λ* is set to 1.

Using the aforementioned method, we conducted experiments on the test set to assess music emotion conversion. Based on high and low-dimensional emotion spaces composed of Arousal and Valence, we performed conversions among four emotions: happy, tense, sad, and calm. The final results were evaluated for accuracy using the music emotion classification model EMPOIA, as shown in the confusion matrix in **Table 4**.

**Table 4:** Evaluation of the accuracy of music emotion transfer.

	T-Happiness	T-Tension	T-Sadness	T-Calmness
S-Happiness	-	60.84%	59.70%	71.56%
S-Tension	63.45%	-	67.81%	57.63%
S-Sadness	58.29%	65.48%	-	64.80%
S-Calmness	70.26%	56.21%	62.35%	-

Overall, transforming latent variables to target emotion clusters using interpolation achieves a certain degree of emotion conversion. This indicates that the Gaussian components for each emotion are well-separated and that each emotion is accurately represented through latent variable information. However, the overall prediction accuracy is not very high, influenced by the EMPOIA classification model, which operates on four emotion categories rather than predicting high and low dimensions of individual emotion dimensions. The experimental results also show that the accuracy of emotion conversion in the Arousal dimension is higher compared to the Valence dimension, with the lowest accuracy observed for conversions across both dimensions. This is consistent with the conclusions in Section 4.4.1, indicating that music is more easily distinguished in the Arousal dimension than in the Valence dimension, and conversions across both dimensions are more challenging. Among the four emotions, the conversion accuracy between happy and calm is the highest, suggesting a high similarity between these two emotions, which allows for effective conversion through changes in rhythmic patterns alone.

## 5. Conclusion

This paper presents the Emotion-Driven Music Generation Model, CDGMVAE, which utilizes GMVAE for semi-supervised clustering inference during training. Compared to fully supervised generative models, CDGMVAE can learn richer data distributions from a small amount of labeled data, effectively mitigating the issue of insufficient emotional music data and enhancing the model’s ability to infer and generate different emotional music categories. To address the mode collapse problem inherent in GMVAE, we analyzed the evidence lower bound and identified the variance regularization term and mutual information suppression term as key contributors. Therefore, we introduced penalties and enhancements for these factors. Experimental results demonstrate that this approach ensures better separation of different emotions in the latent space, strengthens the correlation between music and emotional information, and improves the robustness and generalization of the semi-supervised model. Given that existing music emotion generation models lack interpretability of emotions, we propose establishing connections between emotions and musical rhythmic and melodic features. By introducing a feature disentanglement mechanism to learn emotional representations of these features and incorporating adversarial loss to enhance feature disentanglement, we achieve controlled manipulation of music emotion transformations. Additionally, we employed Transformer-XL as the encoder and decoder for GMVAE, which effectively learns longer contextual dependencies in music sequences, further enhancing the realism of the generated music.

## Supporting information

S1 Text Supplementary experimental results. (DOCX)
