# Supplementary material for: Semi-supervised emotion-driven music generation model based on category-dispersed Gaussian Mixture Variational Autoencoders
Source: PLoS One. 2024 Dec 30;19(12):e0311541. doi: 10.1371/journal.pone.0311541 (PMC11684628; doi:10.1371/journal.pone.0311541)
Supplement: S1 Text — (DOCX) [file pone.0311541.s001.docx]

**Supplementary experimental results**

To more clearly observe the changes in music after altering feature representations, two pieces of music, A and B, were randomly selected from the test set and input into the best model, Semg-GMVAE, for a controlled generation evaluation from a different perspective. The control method remained the same, involving the swapping of the feature representations of the two pieces. However, the generated samples were compared using the piano roll matrix and pitch histogram. The piano roll matrix is a two-dimensional matrix where the horizontal axis represents time steps in beats, and the vertical axis represents the pitch of all notes played at a given moment, reflecting changes in notes overall. The open-source tool pypianoroll was used to extract the piano roll matrix of the main melody track over 8 bars, and the data were visualized.

The pitch histogram, which reflects the tonal characteristics, is a data structure representing the key signature of the music. The horizontal axis represents 12 different pitch classes, and the vertical axis shows the frequency of occurrence for each class. The pitch histogram was calculated using the open-source tool PrettyMIDI. Figures 1 and 2 show the piano roll matrix and pitch histogram of the original music samples A and B, respectively. From these figures, it can be observed that sample A exhibits a higher note density in each bar, with a faster rhythm and an overall key of G minor, representing the emotional state of A1-V0. In contrast, sample B has a lower note density, a more relaxed rhythm, and an overall key of C# minor, corresponding to the emotional state of A0-V1.

After swapping the rhythmic feature representations of A and B, two new music samples were generated. Their piano roll matrices and pitch histograms are shown in Figures 3 and 4. The results demonstrate that when A adopted B's rhythmic features, the generated music captured B's rhythm, exhibiting lower note density, longer note durations, and less variation overall. Conversely, B became more dynamic and compact after incorporating A's rhythmic features. Notably, since only the rhythmic features were swapped, the tonal characteristics of both samples remained relatively unchanged, resulting in a shift in emotional expression along the Arousal dimension.

On the other hand, when the tonal feature representations of A and B were exchanged, similar outcomes were observed, as shown in Figures 5 and 6. While their rhythmic patterns remained intact, the tonalities were swapped after learning from each other, leading to changes in emotional expression along the Valence dimension.


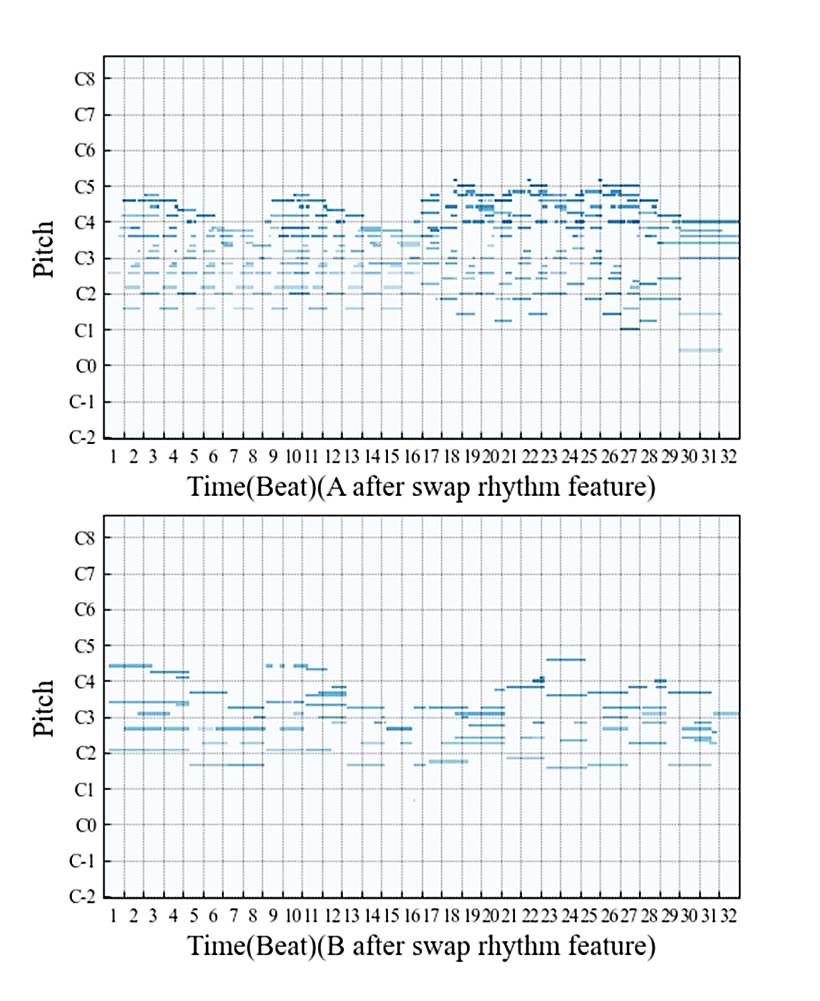


**Figure 1**. Piano roll matrices of original music samples A and B.


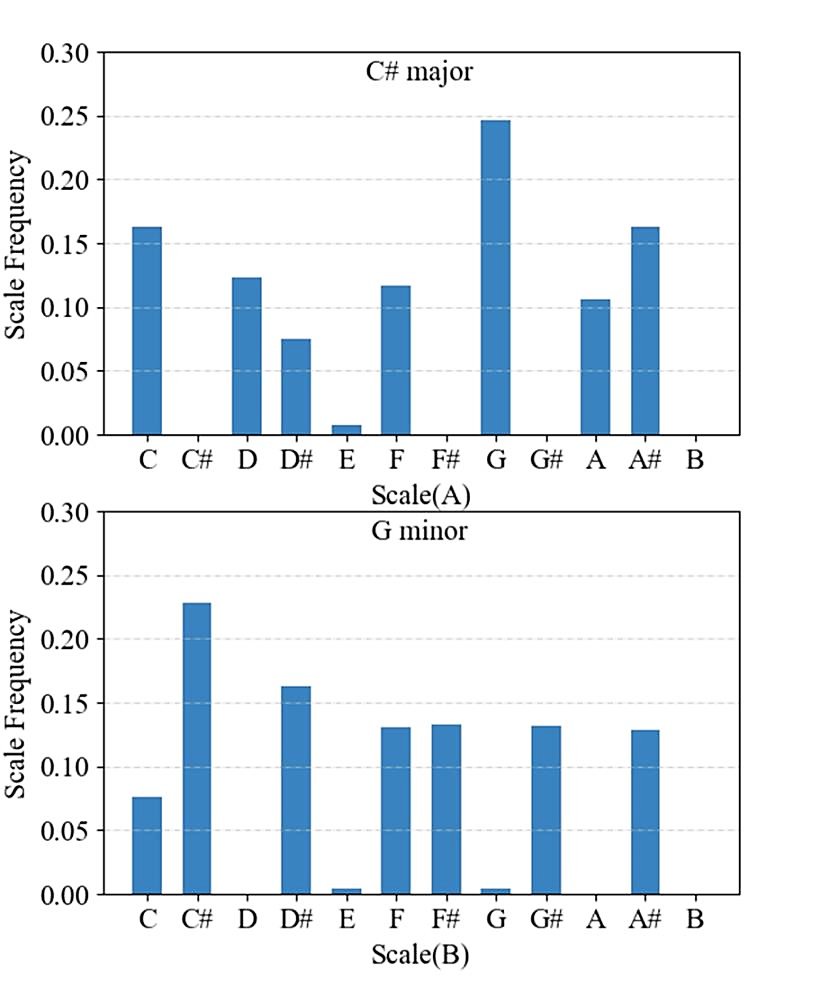


**Figure 2**. Pitch histograms of original music samples A and B.


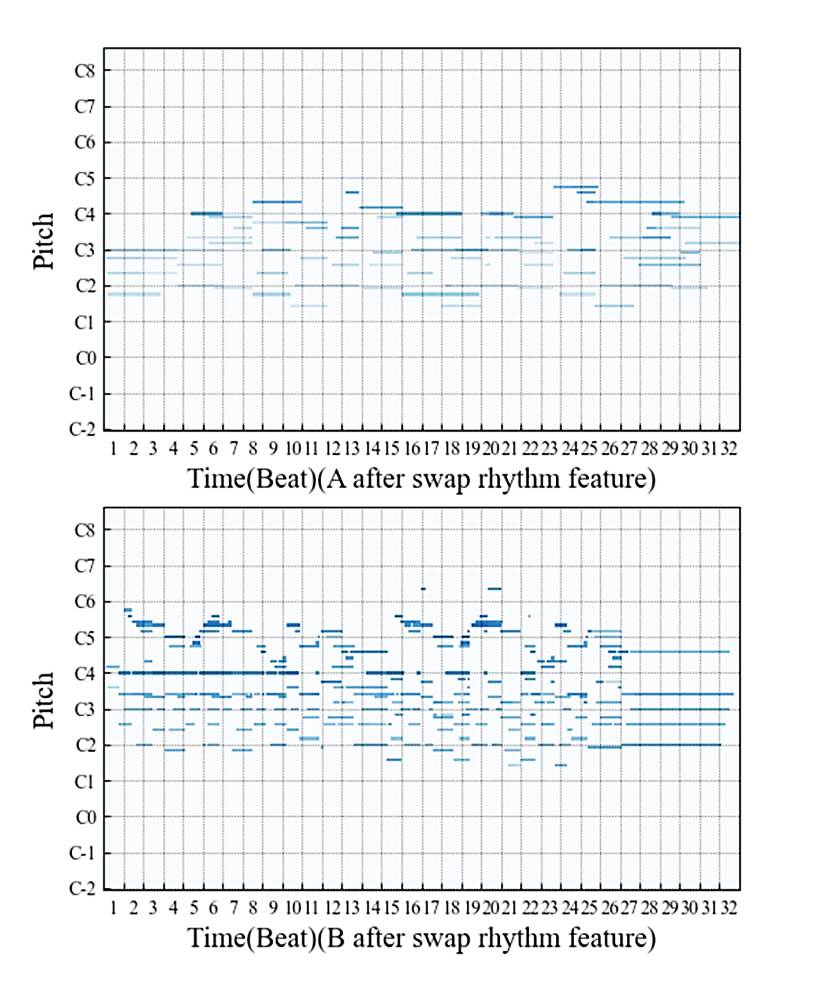


**Figure 3**. Piano roll matrix of music samples A and B after exchanging rhythmic feature representations.


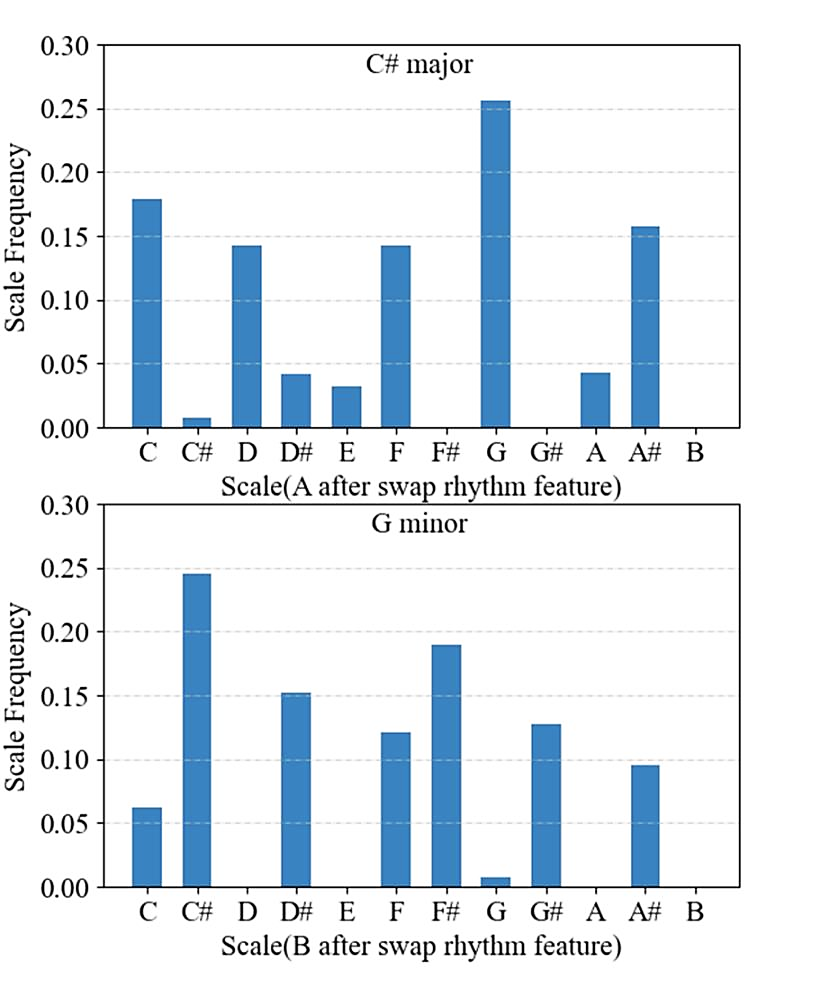


**Figure 4**. Pitch histogram of music samples A and B after exchanging rhythmic feature representations.


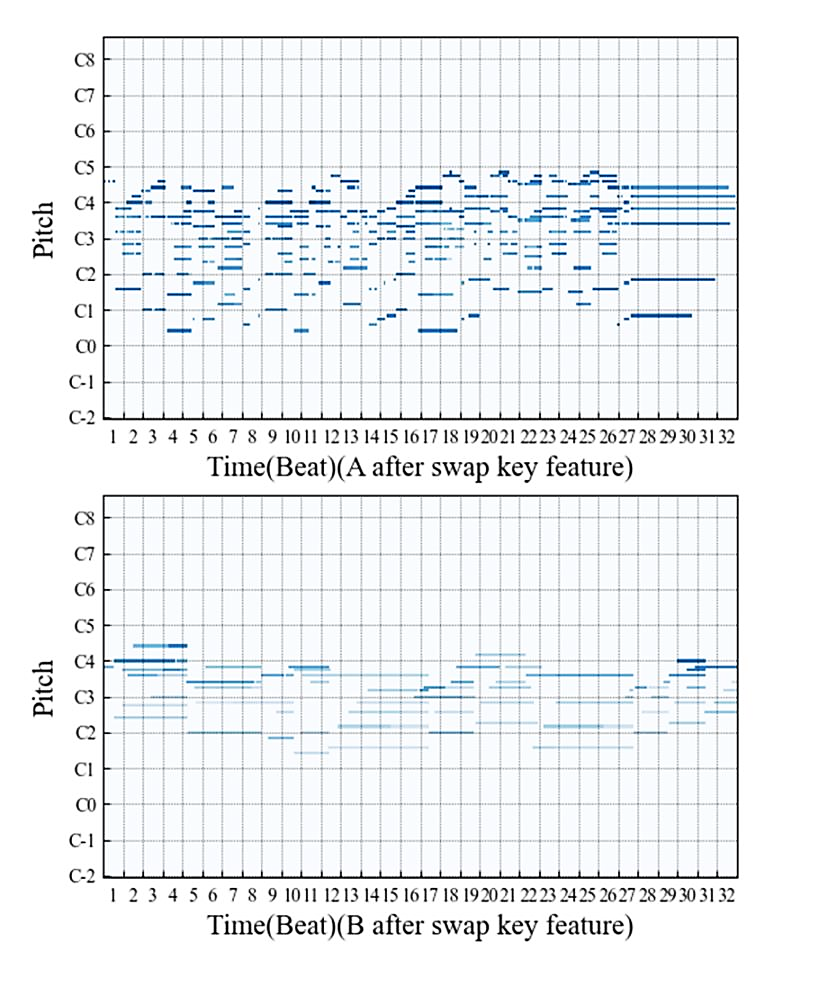


**Figure 5**. Piano roll matrix of music samples A and B after exchanging tonality feature representations.


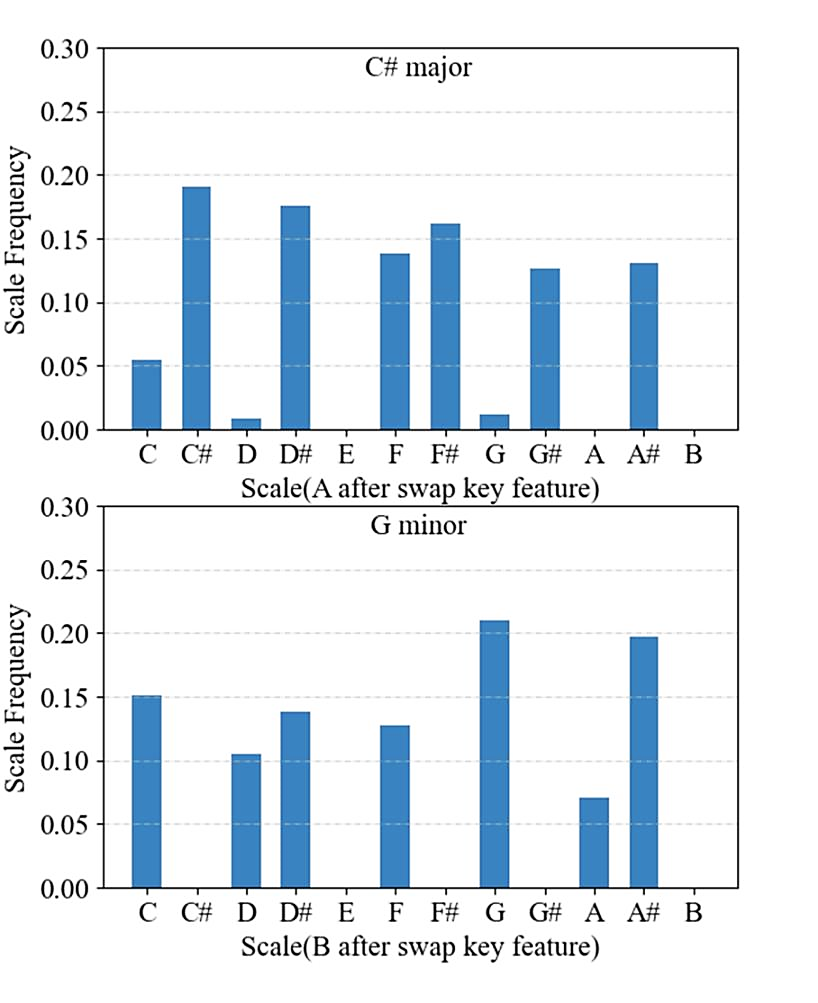


**Figure 6**. Pitch histogram of music samples A and B after exchanging tonality feature representations.
